# Enhanced mechanical, thermal and biocompatible nature of dual component electrospun nanocomposite for bone tissue engineering

**DOI:** 10.7717/peerj.6986

**Published:** 2019-05-27

**Authors:** Guanbao Li, Pinquan Li, Qiuan Chen, Mohan Prasath Mani, Saravana Kumar Jaganathan

**Affiliations:** 1 Department of Minimally Invasive Spine Surgery, Yulin City Orthopaedic Hospital of Traditional Chinese Medicine and Western Medicine, Yulin City, Guangxi, China; 2 School of Biomedical Engineering and Health Sciences, Faculty of Engineering, Universiti Teknologi Malaysia, Skudai, Malaysia; 3 Department for Management of Science and Technology Development, Ton Duc Thang University, Ho Chi Minh City, Vietnam; 4 Faculty of Applied Sciences, Ton Duc Thang University, Ho Chi Minh City, Vietnam; 5 IJNUTM Cardiovascular Engineering Center, School of Biomedical Engineering and Health Sciences, Faculty of Engineering, Universiti Teknologi Malaysia, Skudai, Malaysia

**Keywords:** Polyurethane, Canola oil/neem oil, Nanocomposite, Tissue engineering, Physico-chemical characteristics, Biocompatibility

## Abstract

Traditionally, in the Asian continent, oils are a widely accepted choice for alleviating bone-related disorders. The design of scaffolds resembling the extracellular matrix (ECM) is of great significance in bone tissue engineering. In this study, a multicomponent polyurethane (PU), canola oil (CO) and neem oil (NO) scaffold was developed using the electrospinning technique. The fabricated nanofibers were subjected to various physicochemical and biological testing to validate its suitability for bone tissue engineering. Morphological analysis of the multicomponent scaffold showed a reduction in fiber diameter (PU/CO—853 ± 141.27 nm and PU/CO/NO—633 ± 137.54 nm) compared to PU (890 ± 116.911 nm). The existence of CO and NO in PU matrix was confirmed by an infrared spectrum (IR) with the formation of hydrogen bond. PU/CO displayed a mean contact angle of 108.7° ± 0.58 while the PU/CO/NO exhibited hydrophilic nature with an angle of 62.33° ± 2.52. The developed multicomponent also exhibited higher thermal stability and increased mechanical strength compared to the pristine PU. Atomic force microscopy (AFM) analysis depicted lower surface roughness for the nanocomposites (PU/CO—389 nm and PU/CO/NO—323 nm) than the pristine PU (576 nm). Blood compatibility investigation displayed the anticoagulant nature of the composites. Cytocompatibility studies revealed the non-toxic nature of the developed composites with human fibroblast cells (HDF) cells. The newly developed porous PU nanocomposite scaffold comprising CO and NO may serve as a potential candidate for bone tissue engineering.

## Introduction

The commonly occurring bone trauma diseases were tissue destruction or bone fracture (Qi et al., 2018). The autograft or allografts are the traditional methods commonly used to treat bone defects. However, they possess certain disadvantages such as donor scarcity, supply limitation, immunogenicity risk and infections which makes their usage limited in bone tissue repairing (Wang & Yeung, 2017). Recently, the development of tissue engineered bone substitutes was widely used in biomedical applications because it overcomes the drawbacks associated with the above traditional methods. Tissue engineering (TE) comprises of three components, namely cells, growth factors and scaffolds (Pereira et al., 2015). Among these, the scaffolds play a vital role in supporting cellular responses and tissue regeneration (Bružauskaite et al., 2016). The scaffold used in the bone tissue engineering should resemble the native extracellular matrix (ECM) structure, and it is still one of the major challenges in the tissue engineering applications (Venugopal et al., 2007). The 3D structure of the natural ECM mainly contains fibrous proteins having a diameter in nano-metric range (Heydarkhan-Hagvall et al., 2008).

Recently, the use of electrospinning is widely reported in the tissue engineering applications which produces the nano-scale matrices. The scaffolds produced using electrospinning technique contains nanofibers which can mimic the native function of the ECM (Wang, Ding & Li, 2013). Electrospinning is a cost-effective and versatile technique which uses high voltage to fabricate the nanofibers from polymeric solution. In electrospinning, there are many parameters are involved like flow rate, the viscosity of the polymer, high voltage and collector distance which may affect the quality of fibers produced (Subbiah et al., 2005). The nanofibers fabricated using electrospinning technique possess high surface area with interconnected pores makes them an attractive choice for biomedical applications (Huang et al., 2003). In this research, the nanofibers were fabricated using Tecoflex EG-80A (medical grade polyurethane). Properties like biodegradability, good barrier properties and better oxidation stability of polyurethane make them as an undisputed choice in tissue engineering applications (Lamba, Woodhouse & Cooper, 1998; Ma et al., 2011).

In this work, a polyurethane nanocomposite comprising canola oil (CO) and neem oil (NO) was fabricated to enhance its biological properties. Traditionally, the use of oils to alleviate the bone-related pain is widely practiced. Generally, the essential oils derived from plants possess anti-inflammatory, antiseptic and antispasmodic properties (Ali et al., 2015). These properties would play a vital role in reducing muscle and joint pain. CO is a vegetable oil derived from the seed of *Brassica napus* which is used as the cooking oil for making food varieties (Dupont et al., 1989). It is considered good for people’s health owing to low saturated and plenty of polyunsaturated fats (Lin et al., 2013). The chemical constituents present in the CO were tocopherols, phytosterols, polyphenols and carotenoids. The active constituents present in the CO were reported to improve oxidative stability and also possess antimicrobial and antioxidant property (Ghazani & Marangoni, 2013). The second candidate employed in this work is NO. Neem or the Margosa trees is generally called as *Azadirachta indica* and the active constituents present in the NO were known for its antimicrobial and numerous medicinal properties. NO contains various active constituents such as nimbin, nimbolide, nimbidin and limonoids (Alzohairy, 2016; Asif, 2012). Further, every part of the neem trees was reported to very useful in the medical field against various human diseases owing to the wide range of its pharmacological attributes (Kumar & Navaratnam, 2013).

The blood compatibility evaluation of the fabricated materials is one of the main factors which decides their role in clinical applications. The developed materials should reduce thrombus formation and less toxic to red blood cells (RBC’s) (Jaganathan & Prasath, 2018). In this study, a novel electrospun bone scaffold based on polyurethane (PU) mixed with CO and NO was fabricated. For the first time, we have investigated the combined effect of these oils in bone tissue engineering. This works aims on the determination of physico-chemical properties, blood compatibility and cyto-compatibility of the newly developed scaffolds to analyze their potential for bone tissue engineering.

## Materials and Methodology

### Materials

Tecoflex EG-80A (medical grade polyurethane) was supplied from Lubrizol and dissolved in dimethylformamide (DMF) solvent (Sigma Aldrich, Dorset, UK). NO (Thurgas Trading, Pulau Pinang, Malaysia) and CO (Naturel, Johor, Malaysia) were procured from the local market. The chemicals such as sodium chloride physiological saline (0.9% w/v) and phosphate buffered saline (PBS) were obtained from Sigma-Aldrich, Kuala Lumpur, Malaysia. Blood compatibility reagents as calcium chloride (0.025 M), rabbit brain activated cephaloplastin and thromboplastin (Factor III) were procured from Diagnostic Enterprises, Solan, India.

### Preparation of solution

PU pellets in DMF was prepared at a concentration of 9 wt% and stirred for 12 h to obtain a homogeneous solution. Similarly, the NO and CO in DMF was prepared at a concentration of 9 wt% and stirred for 1 h maximum. The PU solution with 9 wt% was mixed with 9 wt% CO at a ratio of 7:2 (v/v%) and the PU (9 wt%) with CO and NO (9 wt%) solution was mixed at a ratio of 7:1:1 (v/v%) respectively. The mixture is stirred for 2 h maximum to attain even dissolution.

### Electrospinning process

**Figure 1:**
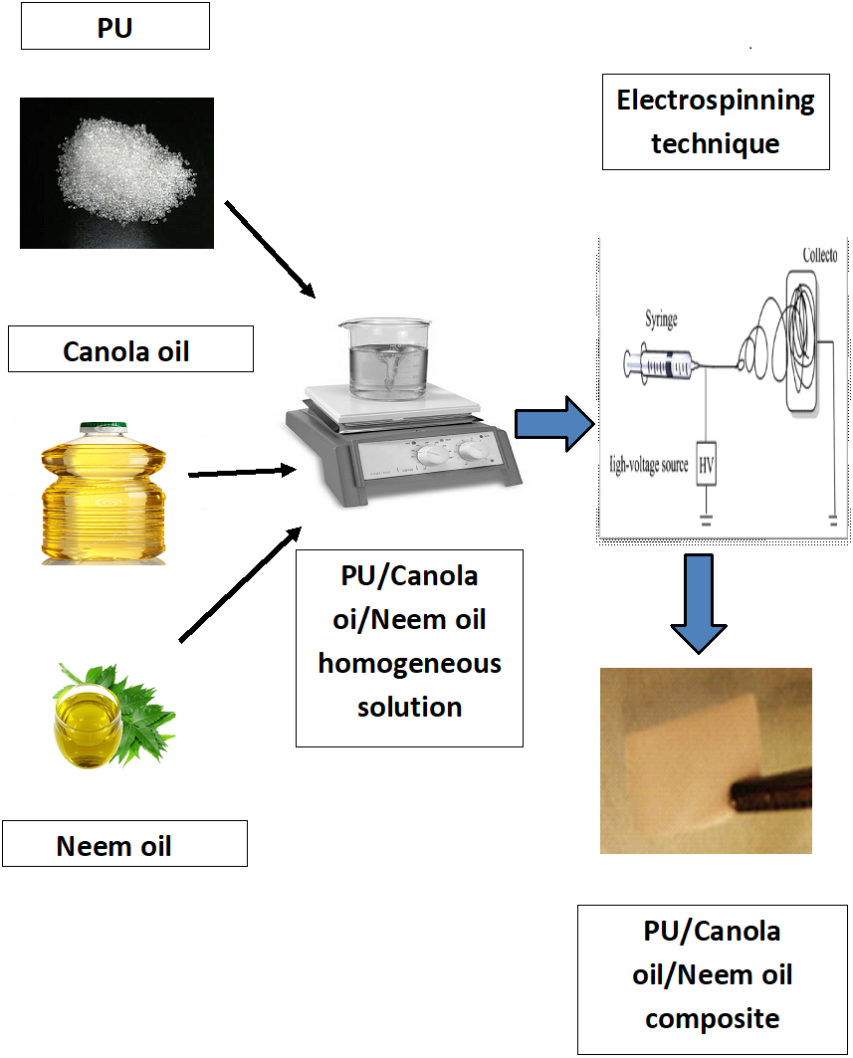
Schematic image involving the materials, preparation of solution and electrospinning process. Images generated by Saravana Kumar Jaganathan.

The electrospinning process was utilized to fabricate the nanofibers. To fabricate the nanofibers, the obtained homogeneous solution is electrospun at conditions of applied voltage 10.5 kV, flow rate of 0.5 ml/h and collector distance 20 cm. The nanofibers collected on the aluminum foil were removed and vacuum dried to eliminate the remaining residual content present in the electrospun membranes. Figure 1 represents the schematic image involving the materials, preparation of solution and electrospinning process.

### Physio-chemical characterizations

#### Scanning electron microscopy

The SEM apparatus (Hitachi Tabletop scanning electron microscopy) was used to investigate the morphologies of the electrospun nanofibrous membrane. The samples were gold plated at 20 mA for 2 min before performing SEM analysis. The analysis was performed at a voltage of 10 kV and the microphotographs were captured at different magnifications. Finally, fiber diameter and its distributions were calculated using Image J by choosing 30 locations randomly from the captured image.

#### IR analysis

The chemical groups in the electrospun membranes were investigated using Fourier-transform infrared (FTIR) analysis through Thermo Nicolet, Waltham, MA. To begin the experiment, a small piece of sample was inspected and the spectra were measured between a wavelength of 600 and 4,000 cm^−1^ with an average of 32 scans per minute at 4 cm^−1^ resolution.

#### Contact angle measurements

The water contact angle measurement determines the surface wettability which was tested using VCA (video contact angle) unit. Initially, the water droplet was placed on the electrospun membrane and the static image of the droplet was captured through a high-resolution video camera. The computer integrated software was used to measure the angle between the water droplet and the surface.

#### Mechanical properties test

The uniaxial load test machine was used to determine the mechanical properties of the electrospun nanofibrous membrane according to ASTM D882-10. All the samples were prepared with a size of 40 mm * 15 mm and gripped at the clamp ends of the tensile machine. The testing was performed with a strain rate of 5 mm/min and load cell of 500 N. The machine record data predict the tensile stress–strain curves and the tensile strength and elongation was calculated from the curve. The gauge length used was 20 mm. The thickness of the electrospun PU, PU/CO and PU/CO/NO were 0.193 mm, 0.086 mm and 0.043 mm while their cross section areas were 2.895 mm^2^, 1.29 mm^2^ and 0.645 mm^2^ respectively.

#### TGA analysis

The thermal properties of the electrospun nanofibers were investigated by the PerkinElmer TGA 4000 unit. A sample weight of about 3 mg was placed on the aluminum and the testing was performed under dry nitrogen atmosphere. A heating rate of 10 °C/min with a temperature range of 30–1,000 °C was applied to the specimen and the data obtained were explored into the excel sheet to draw the TGA curves and derivative weight loss curves.

#### Surface measurement analysis

Quantitative surface roughness analysis of the electrospun nanofibrous membrane was investigated by atomic force microscopy (AFM) unit (Nanowizard; JPK Instruments). To begin, the samples are fixed on a specimen holder and the scanning is performed at room temperature in a normal atmosphere. The scanning was performed in a 20 µm × 20 µm area by tapping mode and the 3D image was obtained with 256 * 256 pixels using JPKSPM data processing software.

### Coagulation assay

#### Activated partial thromboplastin time (APTT) and Prothrombin time (PT) assay

To evaluate the antithrombogenicity of the developed nanofibrous mats, the coagulation assays APTT and PT were utilized. Activated partial thromboplastin time is an intrinsic pathway to measure the time taken for the activation of the blood clot. To start the assay, the fabricated electrospun membranes were incubated with PBS at 37 °C for 30 min. After incubation, the samples were added with 50 µl of platelet-poor plasma (PPP), followed by adding 50 µL of the APTT agent (rabbit brain activated cephaloplastin) and incubated at 37 °C for 3 min respectively. Further, 50 µL of calcium chloride (CaCl_2_) (0.025 M) solution was added to the above mixture which initiates the blood and APTT were measured. Similarly, the prothrombin assay was also done to measure the time taken for blood clot through the extrinsic pathway. For the PT assay, the steps involved were similar to the APTT assay. For PT assay, the samples were added with 50 µl of PPP, followed by the adding 50 µL of the PT agent (thromboplastin (Factor III)) respectively. To initiate the blood clot, the mixture was stirred using the needle and the PT was measured (Manikandan et al., 2017).

#### Hemolysis assay

Hemolysis assay was carried out to analyze the toxicity of fabricated membranes against red blood cells (RBCs). Initially, the mixture of citrated blood and diluted saline was done at a ratio of 4,5 (v/v%). Next, the sample with a size of (1 × 1 cm^2^) was cut and was soaked in physiological saline at 37 °C for 30 min. After 30 min, the samples added to the prepared mixture of citrated blood and diluted saline for 1 h at 37 °C. Then, the samples were detached and centrifuged at 3,000 rpm for 15 min. Finally, the optical density (OD) was measured from the aspirated supernatant at 542 nm to evaluate the hemoglobin release. The percentage of hemolysis or hemolytic index was calculated as described earlier (Manikandan et al., 2017).

#### Cytocompatibility studies

Human Dermal Fibroblast (HDF) cells were cultured in the medium of Dulbecco’s Modified Eagle Medium (DMEM) supplemented with 10% fetal bovine serum and incubated with 5% carbon dioxide (CO_2_) at 37 °C. For every 3 days, the medium was changed. Prior to the cell seeding, the prepared electrospun scaffolds were cut into the small size and placed in 96-well plates. The electrospun membrane placed in the culture plates were sterilized with 75% alcohol solution for 3 h and washed with PBS. Then, HDF cells with a density of 10 × 10^3^ cells/cm^2^ were placed on each electrospun membrane and fixed in CO_2_ incubator. After 3 days culturing, the cellular electrospun mats were added with 20% of 3-(4,5-dimethylthiazol-2-yl)-5-(3-carboxymethoxyphenyl)-2-(4-sulfophenyl)-2H tetrazolium, inner salt (MTS) and incubated for 4 h. After 4 h, the culture plates were retrieved and OD was measured at 490 nm using spectrophotometric plate reader to determine the viability rates.

#### Statistical analysis

One way ANOVA followed by Dunnett post hoc test was performed for experiments results with 3 trails to determine statistical significance. The analyzed data were expressed as mean ± SD. A representative of three images was presented for qualitative experiments.

## Results

### SEM investigation

**Figure 2:**
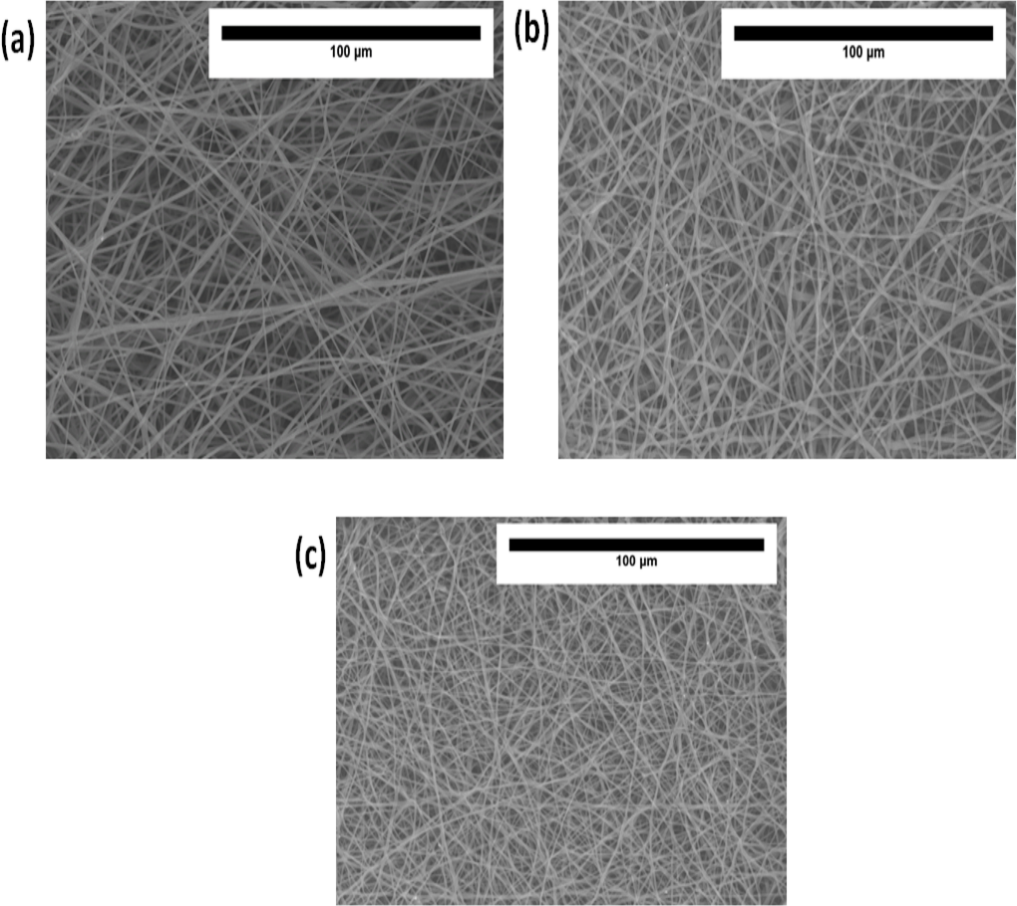
SEM photographs of (A) polyurethane, (B) polyurethane/CO composites, and (C) polyurethane/CO/NO composites. Sample with size of 1 cm * 1 cm was cut and imaged in Hitachi Tabletop TM3000.

**Figure 3:**
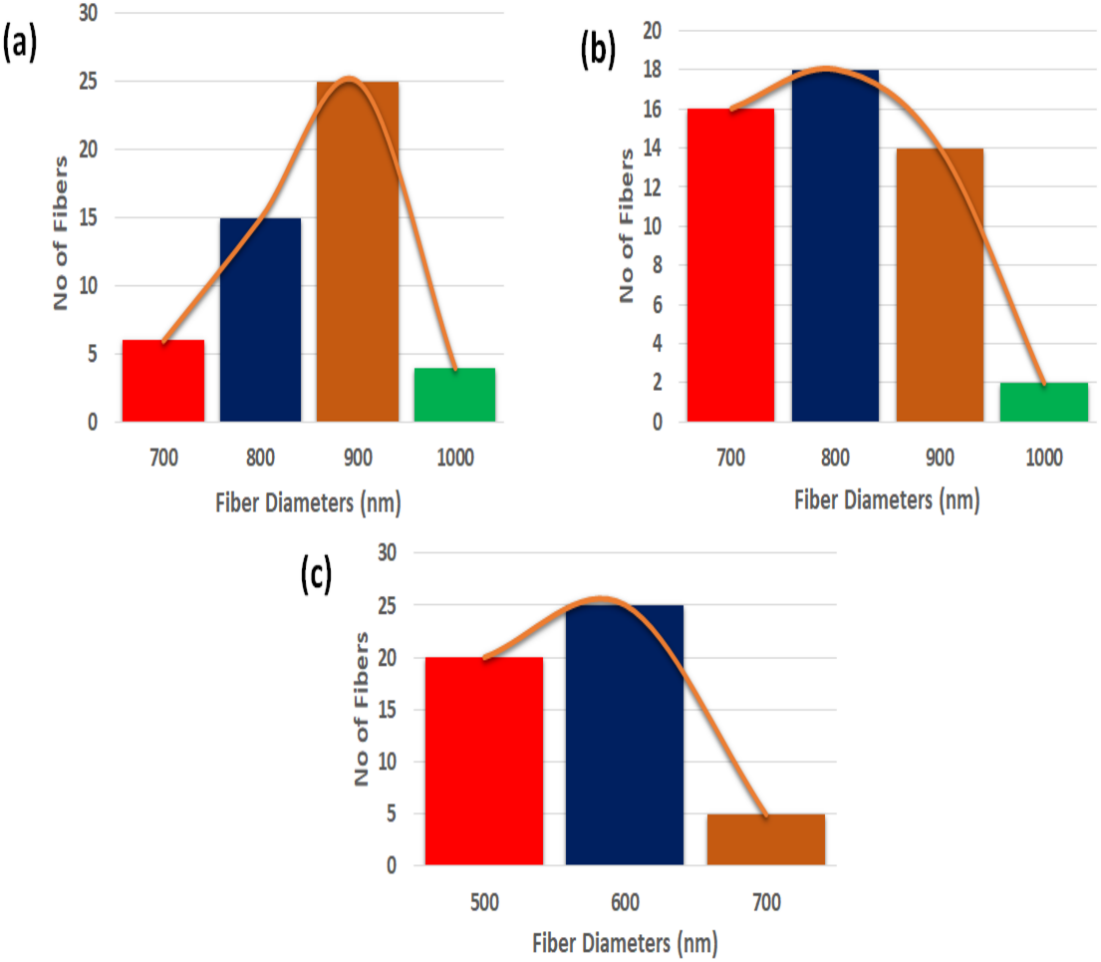
Fiber diameter distribution of (A) polyurethane, (B) polyurethane/CO composites, and (C) polyurethane/CO/NO composites. Measured using Image J software by choosing 50 locations randomly.

Figures 2A–2C indicate SEM images of PU and their nanocomposites fabricated with CO and NO. SEM images depicted that the fibers of electrospun PU, PU/CO and PU/CO/NO membranes were randomly oriented and rendered beadles morphology. The fiber diameters of PU, PU/CO scaffold, PU/CO/NO scaffold were estimated as 890 ± 116.911 nm, 853 ± 141.27 nm and 633 ± 137.54 nm and their fiber distribution curve as shown in Figs. 3A–3C.

### IR analysis

**Figure 4:**
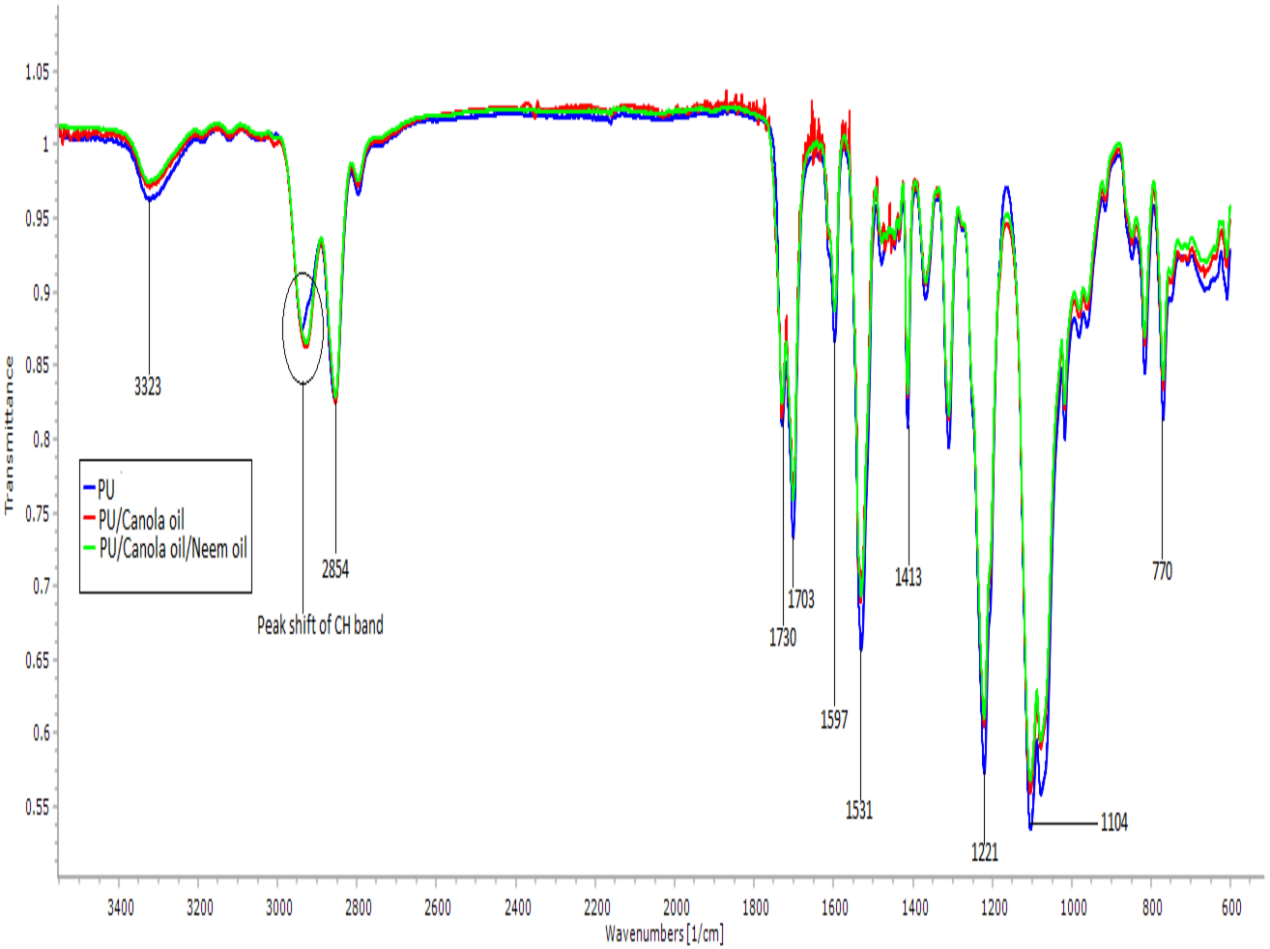
IR spectrum of polyurethane, polyurethane/CO composites, and polyurethane/CO/NO composites. Sample with size of 1 cm * 1 cm was cut and measured in wavelength range between 600 and 4,000 cm^−1^ in Nicolet iS5.

Attenuated total reflectance-Fourier-transform infrared (ATR-FTIR) analysis was carried out to identify the characteristic peaks present in the fabricated PU, PU/CO and PU/CO/NO membranes as presented in Fig. 4. The spectra of PU membrane include 3,323 cm^−1^ (NH stretching), 2,939 cm^−1^ and 2,854 cm^−1^ (CH stretching), 1,730 cm^−1^ and 1,703 cm^−1^ (carbonyl stretching), 1,597 cm^−1^ and 1,531 cm^−1^ (vibrations of NH stretching), 1,413 cm^−1^ (vibrations of CH_2_ stretching) and 1,221 cm^−1^ and 1,104 cm^−1^ (CO stretch corresponding to alcohol group) (Manikandan et al., 2017). In the spectra of PU/CO and PU/CO/NO scaffolds, the peaks were similar; however, decrease in peak intensity was found with the addition of canola and neem oil. Further, the electrospun nanocomposites exhibited a peak shift of CH stretch in pure PU from 2,939 cm^−1^ to 2,930 cm^−1^ in PU/CO and 2,929 cm^−1^ in PU/CO/NO scaffold.

### Wettability measurements

The results of water wettability for the developed PU, PU/CO and PU/CO/NO scaffolds were listed in Table 1. The results of water wettability test indicated that PU scaffold exhibited the contact angle of 100° ± 0.58 and for electrospun PU/CO scaffold, it was increased to 108.7° ± 0.58 and reduced to 62.33° ± 2.52 in PU/ CO/NO scaffold.

### Thermal analysis

**Figure 5:**
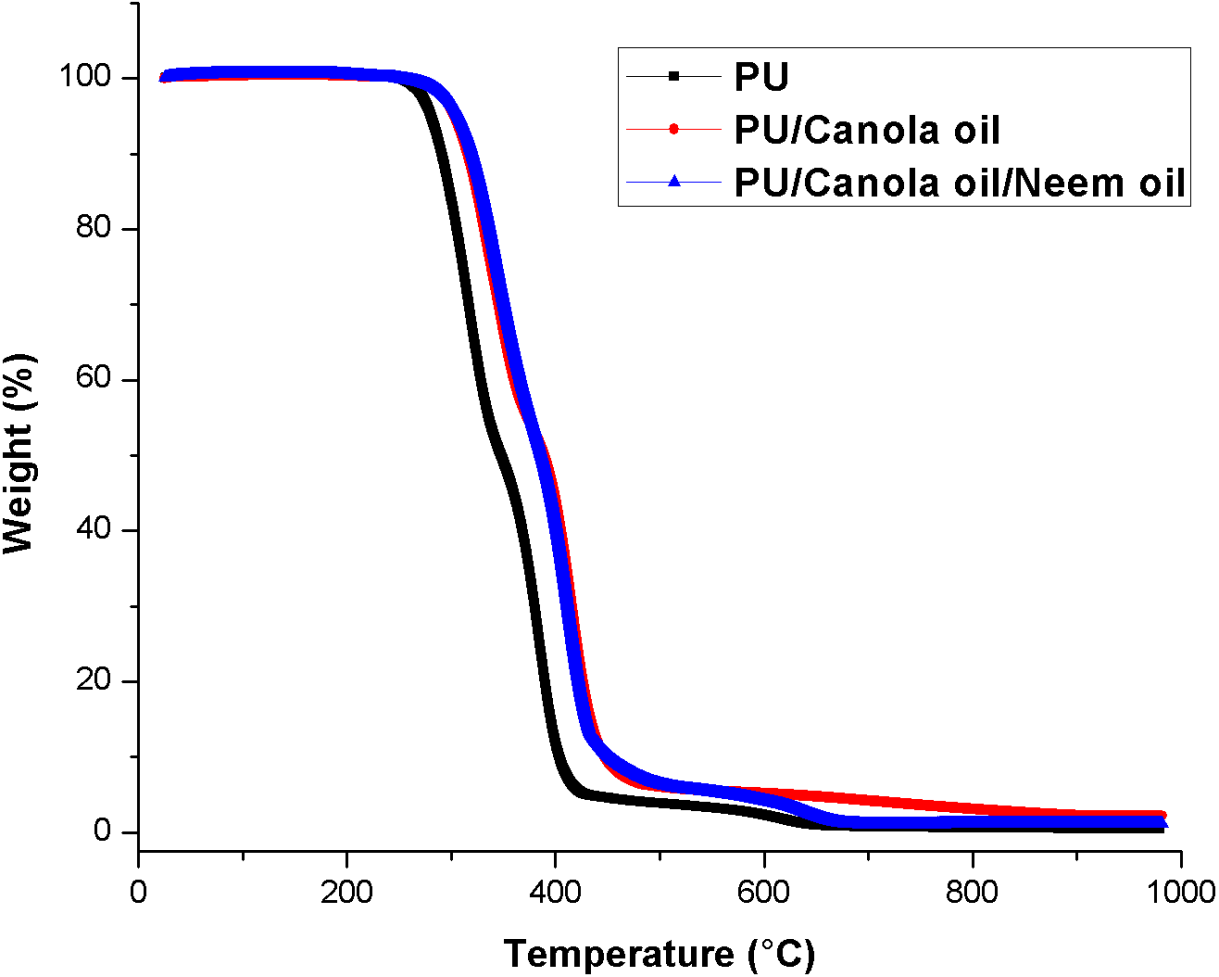
Thermal behavior of polyurethane, polyurethane/CO composites and polyurethane/CO/NO composites. Sample weighing 3 mg was heated between temperature range of 30–1,000 °C under nitrogen atmosphere in Perkin-Elmer TGA unit.

**Figure 6:**
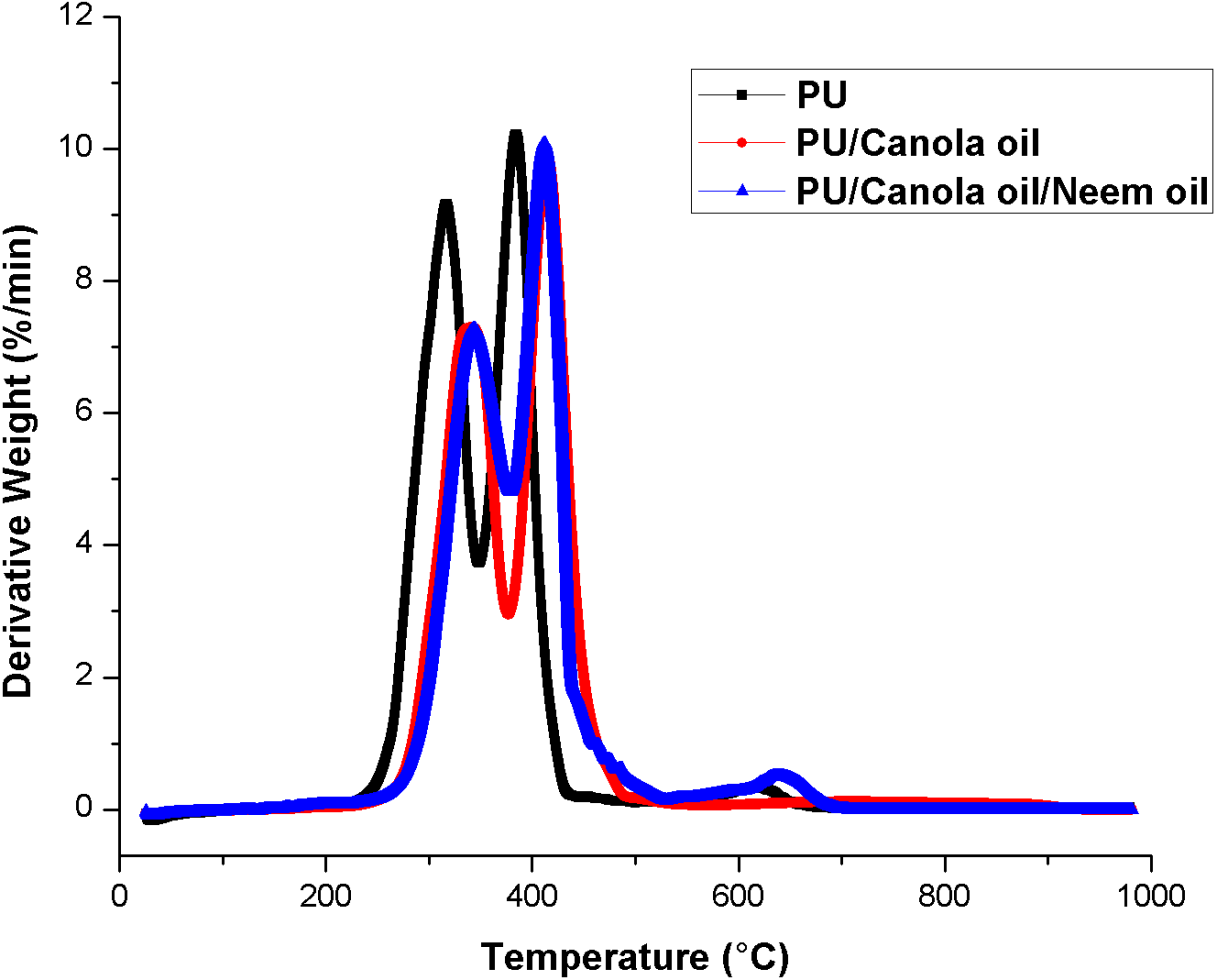
DTG curve of polyurethane, polyurethane/CO composites and polyurethane/CO/NO composites. Sample weighing 3 mg was heated between temperature range of 30–1,000 °C under nitrogen atmosphere in Perkin-Elmer TGA unit.

The results of the thermal analysis for developed PU, PU/CO and PU/CO/NO scaffolds were presented in Figs. 5A–5C. TGA demonstrated the initial temperature decomposition of PU membrane starts at 276 °C, while for the developed PU/CO and PU/ CO/NO scaffold, the initial decomposition temperature was 296 °C and 306 °C respectively. At 1,000 °C, the residue weight percentage of PU was found to be only 0.47%, whereas for the developed PU/CO and PU/ CO/NO scaffold were observed to be 2.23% and 1.22% respectively indicating the interaction of PU with CO and NO. Further, the DTG curve of PU/CO and PU/CO/NO scaffold was shown in Figs. 6A–6C. In PU, there was three weight loss namely two major loss and one minor loss. The first and second major loss occurs at 223 °C to 348 °C and 348 °C to 446 °C, while the third minor loss was seen at 557 °C to 684 °C respectively. In the case of PU/CO scaffold, it shows two losses only, the first weight loss at 234 °C to 377 °C and final loss at 377 °C to 545 °C. While in the PU/CO/NO scaffold, it showed three weight loss in which starts from 223 °C to 378 °C (first loss), 378 °C to 529 °C (second loss) and 529 °C to 697 °C (third loss) respectively.

**Table 1:** Contact angle Measurement of PU, PU/CO and PU/CO/NO composites. Sample with size of 1 cm * 1 cm was cut and a water droplet of size 0.5 µl was placed on it. The static image of the droplet was captured through a high-resolution video camera in VCA equipment and the manual contact angle was determined.

**S.No**	**Sample**	**Average contact angle in degrees**
1	Pure Polyurethane	100° ± 0.5774
2	Polyurethane/CO composites	108.7° ± 0.5774^*^
3	Polyurethane/CO/NO composites	62.33° ± 2.517^*^

**Notes.**

* mean differences were significant compared with pure PU (*p* < 0.05).

### Surface measurements

**Figure 7:**
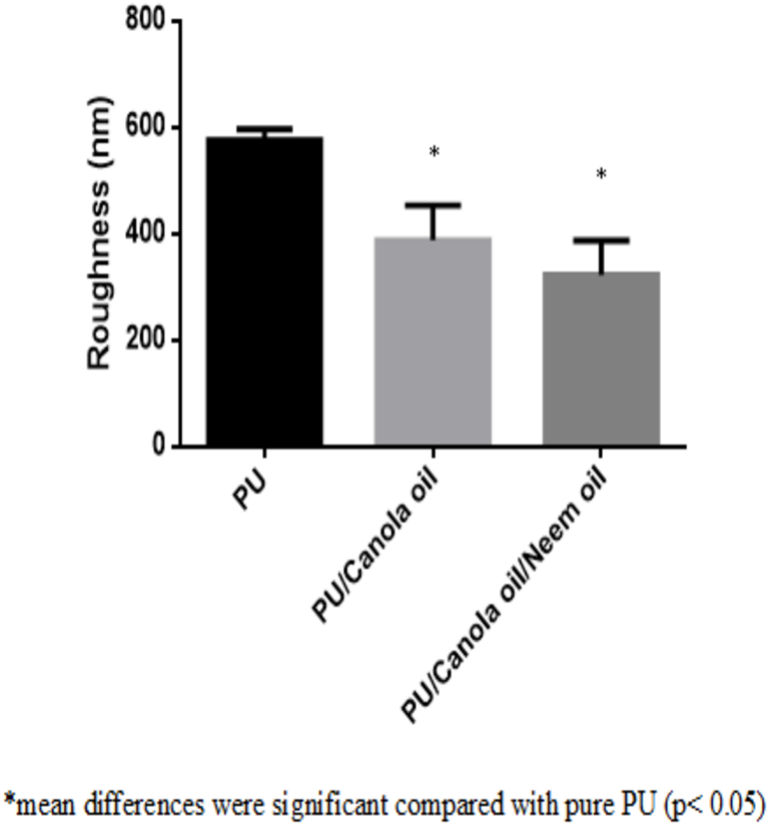
Surface measurements of polyurethane, polyurethane/CO composites, polyurethane/CO/NO composites. Sample with size of 1 cm * 1 cm was cut and scanned in 20 * 20 µm size with 256 * 256 pixels under normal atmosphere in Nanowizard, JPK instruments.

The measurement of surface roughness for developed PU, PU/CO and PU/CO/NO scaffold were shown in Figs. 7A–7C. The average roughness of PU membrane was found to be 576 nm, while the electrospun PU/CO/NO and PU/CO/NO scaffold exhibited roughness of 389 nm and 323 nm respectively.

### Tensile analysis

**Figure 8:**
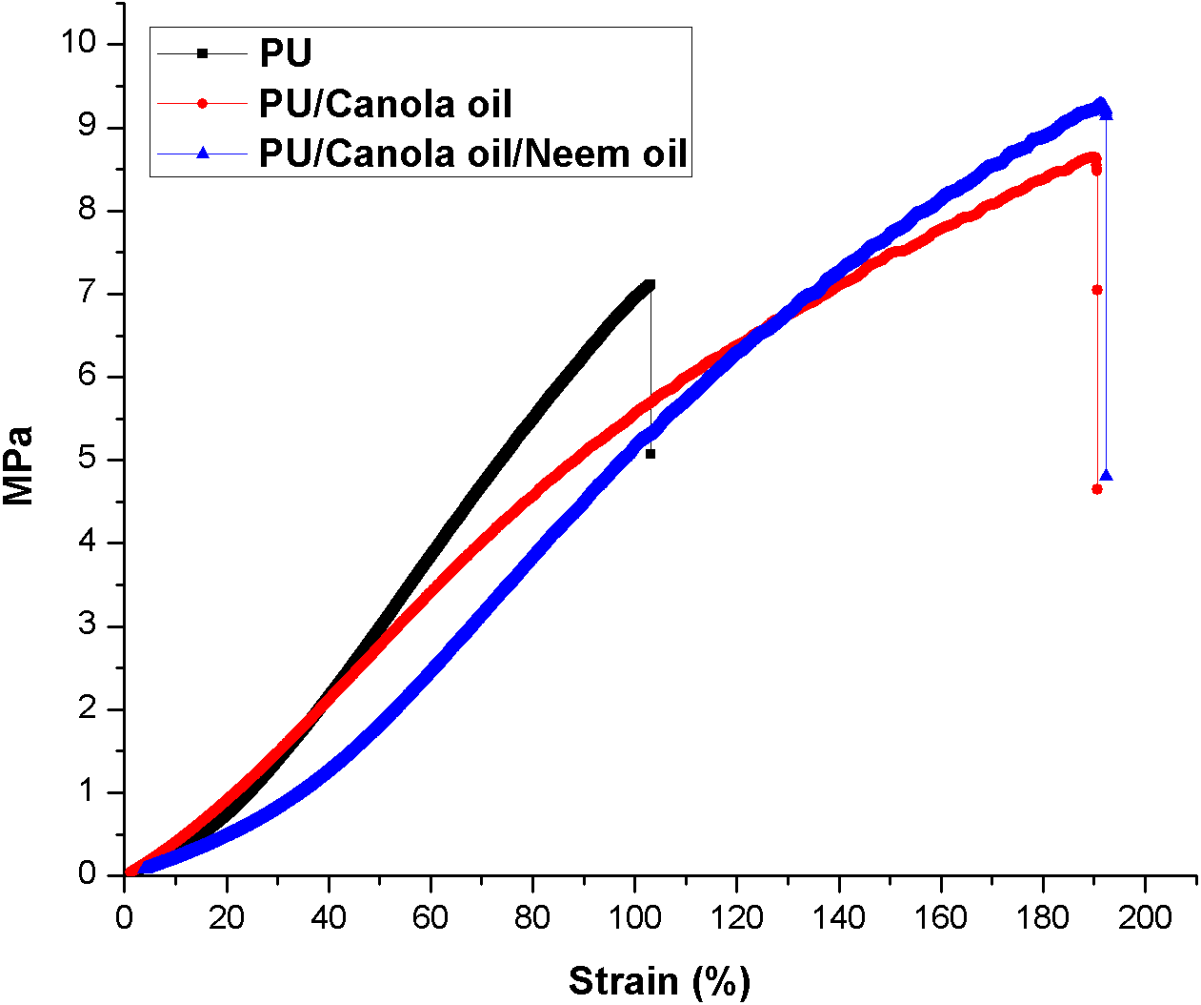
Tensile strength of polyurethane, polyurethane/CO composites and polyurethane/CO/NO composites. Sample with size of 4 cm * 1 .5 cm was stretched at a cross head speed of 5 mm/min with a 500 N load cell in Gotech Testing Machines, AI-3000.

The stress strain curves of the developed PU, PU/CO and PU/CO/NO scaffold were presented in Figs. 8A–8C. The PU nanofibers exhibited the tensile strength of 7.373 ± 0.4917 MPa with elastic modulus of 7.033 ± 2.129 MPa. The tensile strength of electrospun PU/CO and PU/CO/NO scaffolds were found to be 8.370 ± 0.2433 MPa and 9.747 ± 0.4061 MPa with elastic modulus of 6.267 ± 0.1155 MPa and 8.497 ± 1.479 MPa respectively.

### Coagulation results

**Figure 9:**
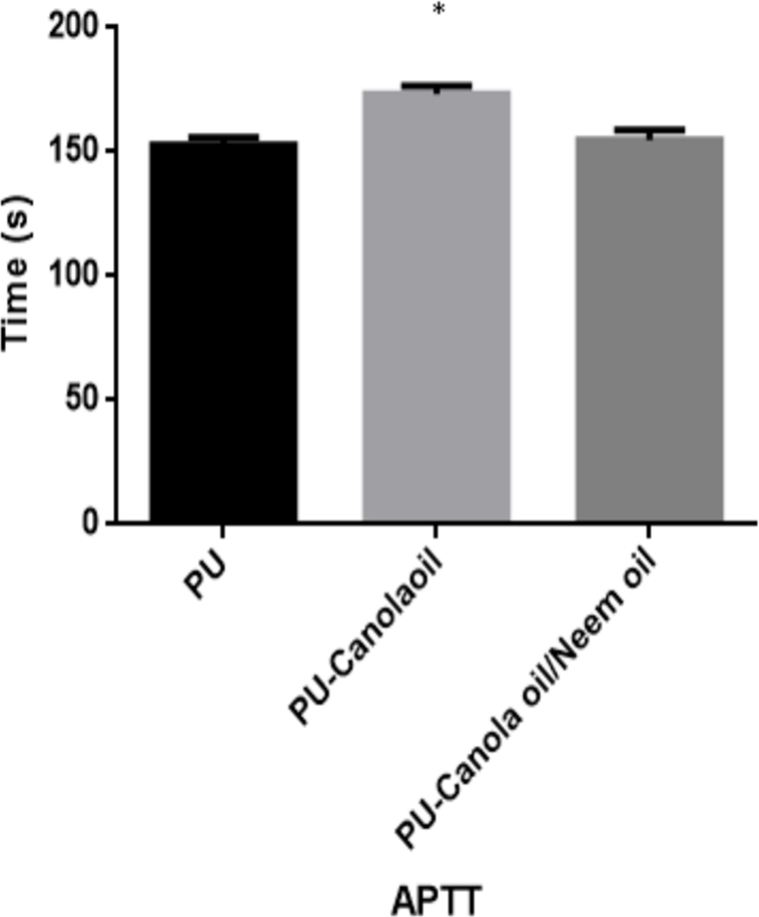
APTT assay of polyurethane, polyurethane/CO composites, and polyurethane/CO/NO composites (mean differences were significant compared with pure PU (*p* < 0.05)). Sample with size of 1 cm * 1 cm was added with 50 µL of platelet-poor plasma (PPP) followed by incubating with 50 µL of reagent (rabbit brain cephaloplastin) and 50 µL CaCl_2_ (0.025 M) to calculate the blood clotting time.

**Figure 10:**
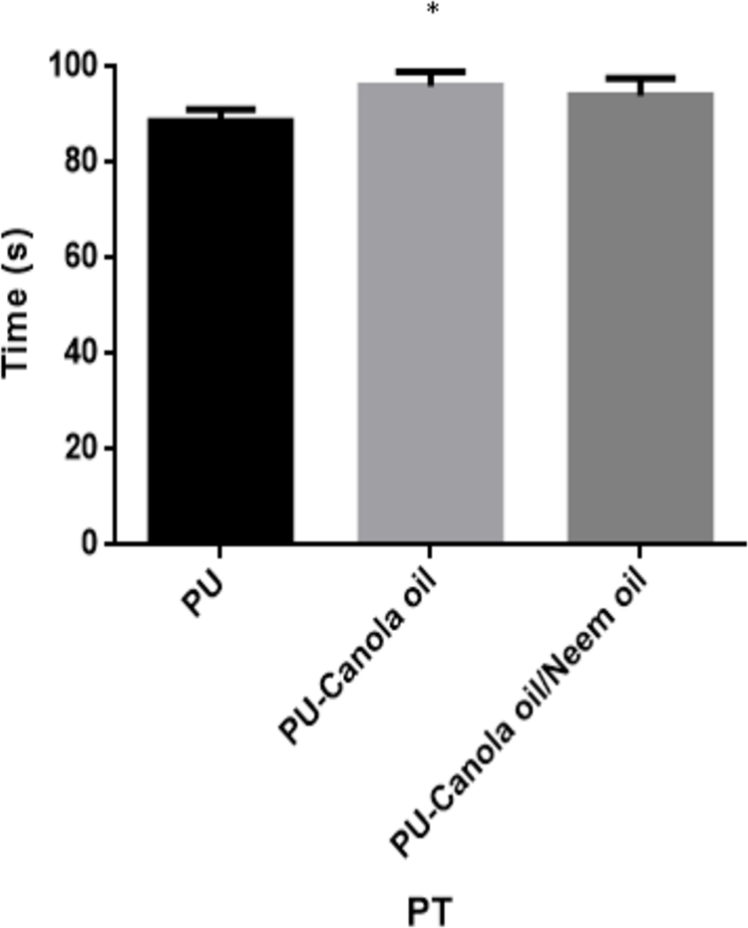
PT assay of polyurethane, polyurethane/CO composites, and polyurethane/CO/NO composites (mean differences were significant compared with pure PU (*p* < 0.05)). Sample with size of 1 cm * 1 cm was added with 50 µL of platelet-poor plasma (PPP) followed by incubating with 50 µL of thromboplastin (Factor III) to calculate the blood clotting time.

**Figure 11:**
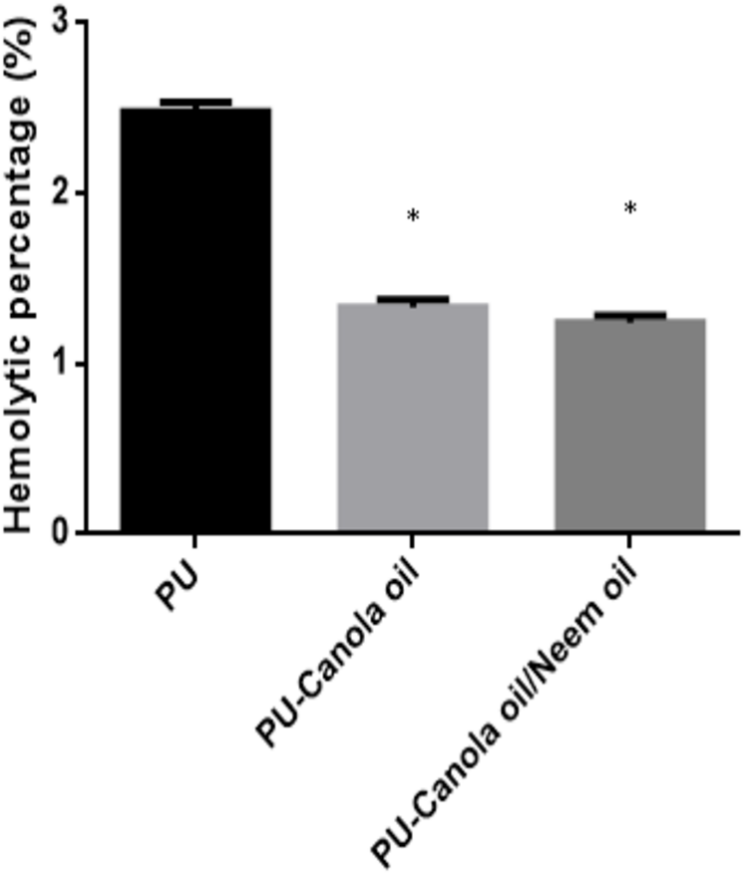
Hemolytic assay of polyurethane, polyurethane/CO composites, and polyurethane/CO/NO composites (mean differences were significant compared with pure PU (*p* < 0.05)). Samples with size of 1 cm * 1 cm added to the mixture of citrated blood and diluted saline (4:5 v/v%) for 1 h at 37 °C. After this, the samples were centrifuged and optical density (OD) was measured at 542 nm.

The APTT and PT of the developed PU, PU/CO and PU/CO/NO scaffold were measured to analyze their clot formation through intrinsic and extrinsic pathway as indicated in Figs. 9 and 10. In APTT assay, the blood clotting of PU membrane was found to 152.7 ± 3.055 s and for electrospun PU/CO and PU/CO/NO scaffold, the blood clotting was observed to be 173.3 ± 3.215 s and 154.7 ± 4.163 s respectively. Similarly, in PT assay, the blood clotting time of PU membrane was found to 88.67 ± 2.517 s and for electrospun PU/CO and PU/CO/NO scaffold, it was observed to 96 ± 3 s and 94 ± 3.606 s respectively. Further, hemolysis assay was done to evaluate the osmotic stress of the developed membranes on the RBCs. For the PU membrane, the hemolytic percentage was found as 2.48% and for the PU/CO and PU/CO/NO scaffold, it was 1.33% and 1.24%, respectively, as indicated in Fig. 11.

### Cell viability analysis

**Figure 12:**
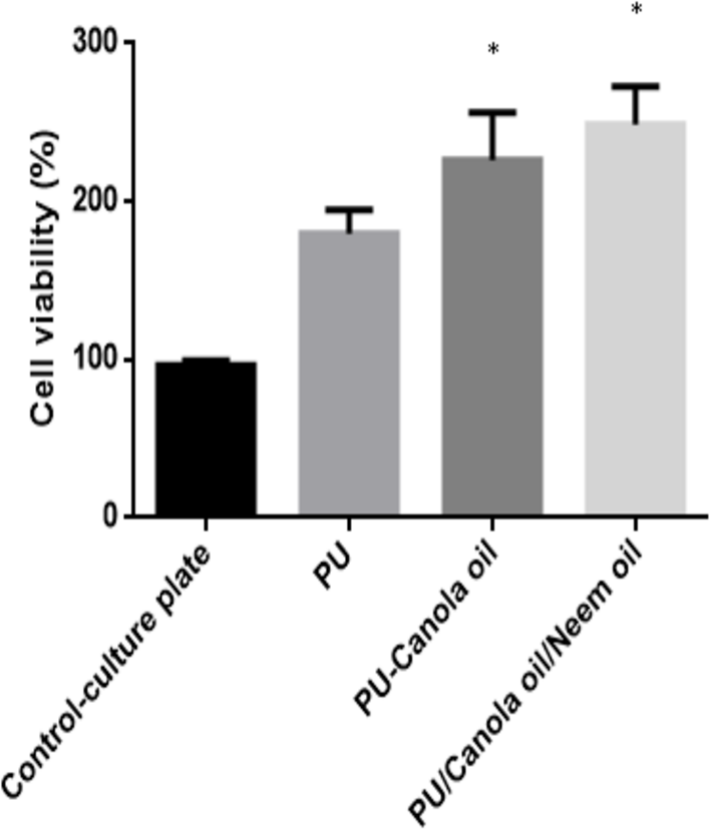
MTS assay of polyurethane, polyurethane/CO composites, and polyurethane/CO/NO composites (mean differences were significant compared with pure PU (*p* < 0.05)). Samples with size of 0.5 cm * 0.5 cm was cut and placed in the 96 well plates. The scaffold were seeded with fibroblast cells with 10 × 10^3^ cells/cm^2^ density and cultured for 3 days. After 3 days, the medium was added with 20% of MTS reagent for 4 h and optical density (OD) was measured at 490 nm.

The nanofibers toxicity was assessed through HDF cells and evaluated after 3 days of cell culturing. Figure 12 indicates the MTS assay results of electrospun PU, PU/canola oil and PU/canola/neem oil scaffold. After 3 days of culture, the cell viability of PU/canola oil and PU/canola/neem oil scaffold were reported to 226.3 ± 29.94% and 248.7 ± 23.97% respectively while the pristine PU membrane exhibited viability rate of 179.7 ± 15.04%.

## Discussion

The performance of the scaffolds used in tissue regeneration mainly depends on certain features like microstructure and host cell responses. Many materials used in tissue engineering possess desirable characteristics but are lacking in blood compatibility or bioactivity. Electrospun PU scaffolds were reported to mimic the ECM matrix but their utilization in tissue regeneration was limited due to poor bioactivity. In this work, CO and NO were added to the electrospun PU fibers to enhance its physico-chemical and cell response. The electrospun PU/CO and PU/CO/NO nanocomposite displayed a reduction in fiber diameter which might be due to the bioactive constituents present in the CO and NO oil. The bioactive constituents may have a putative role in altering the solution parameters which would have resulted in the reduced fiber diameter. A similar effect was observed in a recent study (Chao, Mani & Jaganathan, 2018). In their work, electrospun PU scaffold added with grape seed oil, honey and propolis for bone tissue engineering. It was observed that the fabricated composites showed a reduction in the fiber diameter than the pristine PU due to the bioactive constituents from the additives. Our developed nanocomposites showed smaller fiber diameter than the pristine PU suggesting its suitability for bone tissue growth. In IR spectrum, the intensity of PU was decreased with the incorporation of canola and neem oil. This was owing to the hydrogen bond formation (Unnithan et al., 2012). Moreover, the prepared nanocomposites showed CH peak shift which concludes the presence of CO and NO content in the PU matrix (Tijing et al., 2012). The wettability analysis revealed that the addition of CO renders the scaffold to be hydrophobic whereas the addition of NO improved the wettability nature. The contact angle which is optimum for adhesion of osteoblast cell is reported to be 0–106° (Wei et al., 2009). Hence, the reported wettability of PU/CO scaffold might reduce the osteoblast cell adhesion. However, the addition of NO into PU/CO scaffold renders hydrophilic nature which lies in the reported wettability which might facilitate the adhesion of osteoblast cells. A scaffold with hydrophilic nature may be suitable for bone tissue engineering applications as depicted in some recent researches. Hassan, Sultana & Hamdan (2014) fabricated poly (ε-caprolactone) scaffold fabricated with hydroxyapatite nanofibers for bone tissue engineering. It was observed that the addition of hydroxyapatite into PCL matrix improve the wettability and suggested hydrophilic surface might enhance the osteoblast growth. To conclude, the ambivalent nature of the developed scaffolds may find potential application in tailoring its wettability to the need. In TGA analysis, the thermal behavior of the PU membrane was enhanced by the addition of CO and NO. Manikandan et al. (2017) developed nanocomposite utilizing polyurethane incorporated with murivennai oil and was reported that the incorporated murivennai oil enhanced the thermal behavior of the pure polyurethane and our findings resembled their observations. The results of DTG depicted addition of CO and NO reduced the weight loss of the PU. The reduced weight loss was observed the decrease in intensity of the weight loss peak of the prepared nanocomposites compared to the pristine PU. The surface measurements suggested the developed nanocomposites exhibited smoother surfaces than the pristine PU. Kim et al. (2016) electrospun poly (ε-caprolactone) scaffolds and investigated the effect of fiber diameter on surface roughness. It was reported that the scaffolds with smaller fiber diameter show the smoother surfaces compared to the larger fiber diameter. Hence, the decrease in the surface roughness of the fabricated composite was might be due to their smaller fiber diameter. Ribeiro et al. (2015) studied the effect of surface roughness on osteoblast cell response in poly (L-lactide) electrospun membranes. It was observed that the electrospun membranes with lower surface roughness showed enhanced osteoblast cell proliferation compared to the higher roughness surfaces. Hence, the reduced surface roughness values of the electrospun nanocomposites might favor the enhanced osteoblast cell adhesion and proliferation. Mechanical results showed that the encapsulation of CO and NO improved the tensile strength of the pristine PU. Few works of literature have been reported that the smaller fiber diameter would result in the improvement of the mechanical strength (Mani et al., 2019; Sheikh et al., 2010). The addition of CO and NO oil resulted in the reduction of the fiber diameter which might have favored the enhancement of the tensile strength. Shanmugavel et al. (2014) fabricated a bone scaffold based on polycaprolactone incorporated with *aloe vera* and silk fibroin. They reported the tensile strength was enhanced by the adding of *aloe vera* and silk fibroin into the PCL matrix. Further, the observed tensile strength of the prepared nanocomposite was found to be 4 MPa and concluded a suitable candidate for bone tissue engineering. Our fabricated electrospun membranes exhibited better tensile strength than those reported values indicating the superiority of the fabricated scaffolds for bone tissue engineering. The blood compatibility assessments revealed the prolonged blood clotting time of the developed nanocomposites. The prolonged blood clotting time was because of the addition of CO and NO into the PU matrix. An important requirement for a scaffold in tissue engineering applications is their ability to retard the thrombus formation. Initially, when CO is introduced in the pristine PU, the surface turns to hydrophobic which facilitate the adhesion of plasma protein irreversibly resulting in prolonged blood clotting times. However, when there is an addition of NO, it introduces surface hydrophilicity in the nanocomposite resulting in blood clotting times reduction than the developed PU/CO scaffolds. This may be due to the trade-off between the polar and apolar regions of the PU/CO/NO scaffold (Szycher, 1991). Strikingly, still the blood compatibility of PU/CO/NO scaffold is still better than the control indicating its suitability in bone tissue engineering application. Recently, Jaganathan et al. (2017) fabricated polyurethane scaffold mixed with castor oil nanofibers and investigated the blood compatibility behavior of the fabricated nanocomposites. They reported that the blood compatibility of the pristine PU was increased with the incorporation of castor oil which resembles our findings. Polyphenols compounds are reported to be found in the castor oil (Chakravartula & Guttarla, 2007). These constituents appear to be one of the components present in CO (Ghazani & Marangoni, 2013) and NO (Nahak & Sahu, 2011). Moreover, the fabricated PU/CO and PU/CO/NO scaffold showed less toxic to RBC. The developed nanocomposites were reported to be non-hemolytic materials because their calculated hemolytic index was below 2% (Manikandan et al., 2017). In the MTS assay, it was observed that the cell viability of the electrospun membranes was enhanced compared to the control plates. The cellular viability of the fabricated composites was better than pure PU. The cell adhesion and proliferation is a multifactorial process and various properties influence it. It have been reported that the cell adhesion is influenced by properties such as fiber diameter (Unnithan et al., 2012), wettability (Miguel et al., 2017), surface roughness (Chou et al., 2009), surface chemistry (Anselme, Ploux & Ponche, 2010) and surface energy (Hallab et al., 2001). Hence, any of these factors would have positively influenced the cell adhesion of the fabricated composites. The addition of NO to the PU/CO scaffold slightly increased the cell adhesion and proliferation. This is because NO rendered the surface to be hydrophilic (62.33°). This value was found to be in the optimal wettability range (40–70°) during which the adhesion and proliferation of fibroblast cells reported to be maximum (Miguel et al., 2017).

## Conclusion

In this work, PU nanocomposites based on CO and NO were electrospun successfully. The fabricated PU nanocomposites showed randomly oriented nanofibers with reduced fiber diameter compared to control. The existence of CO and NO in PU matrix was confirmed by the formation of hydrogen bond. The developed PU/CO scaffold rendered hydrophobic while the PU/CO/NO scaffold exhibited hydrophilic behavior and both scaffolds exhibited enhanced thermal stability spotted than the pristine PU. The surface roughness of PU/CO and PU/CO/NO scaffold were found to reduce compared to pure PU. Further, the developed nanocomposites showed enhanced blood compatibility behavior than the pristine PU. Moreover, the newly electrospun scaffolds were observed to non-toxic against RBC and HDF cells than the pristine PU as noted in the hemolytic assay and MTS assay. Finally, this research suggests that the novel developed PU nanocomposite comprising CO and NO with better physio-chemical characteristics, improves blood compatibility behavior and non-toxic nature may be beneficial for repairing the bone defects caused by trauma.

##  Supplemental Information

10.7717/peerj.6986/supp-1 Dataset S1 Raw data for AFM analysis

10.7717/peerj.6986/supp-2 Dataset S2 Raw data for FTIR analysis

10.7717/peerj.6986/supp-3 Dataset S3 Raw data for tensile analysis

10.7717/peerj.6986/supp-4 Dataset S4 Raw data for TGA analysis

10.7717/peerj.6986/supp-5 Dataset S5 Raw data for morphology analysis

## Additional Information and Declarations

### Data Availability

The following information was supplied regarding data availability: Raw data are available as Supplemental Files.
