# Supplementary material for: Enhanced mechanical, thermal and biocompatible nature of dual component electrospun nanocomposite for bone tissue engineering
Source: PeerJ. 2019 May 27;7:e6986. doi: 10.7717/peerj.6986 (PMC6542347; doi:10.7717/peerj.6986)
Supplement: Dataset S5 [file peerj-07-6986-s005.zip › SEM/PU Canola Neem oil/Electron Image.docx]

**Electron Image**

Project: New project

Input Source: Secondary electron detector

Image Width: 181.2 µm


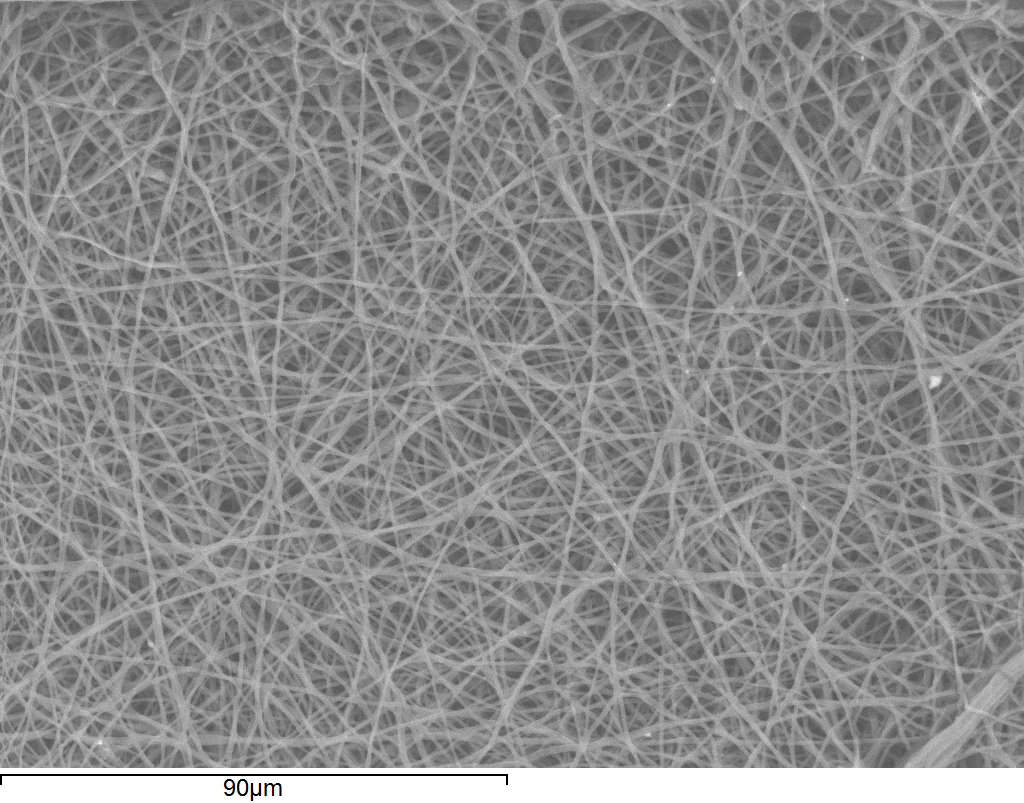


**Spectrum details**

Project New project

Spectrum name Spectrum1

**Acquisition conditions**

Acquisition time (s) 15.0

Process time 5

Accelerating voltage (kV) 15.0


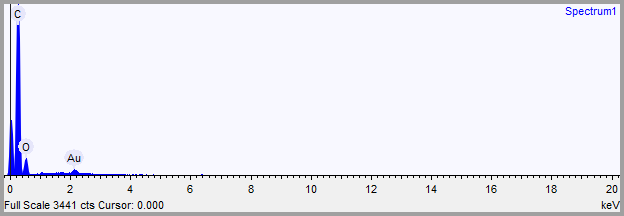


**Quantification Settings**

Quantification method All elements (normalised)

Coating element None

**Summary results**

| Element | Weight % | Weight % σ | Atomic % |
| --- | --- | --- | --- |
| Carbon | 74.960 | 0.771 | 81.243 |
| Oxygen | 22.877 | 0.753 | 18.614 |
| Gold | 2.163 | 0.299 | 0.143 |
