# Supplementary figures and images for: Enhanced mechanical, thermal and biocompatible nature of dual component electrospun nanocomposite for bone tissue engineering
Source: PeerJ. 2019 May 27;7:e6986. doi: 10.7717/peerj.6986 (PMC6542347; doi:10.7717/peerj.6986)

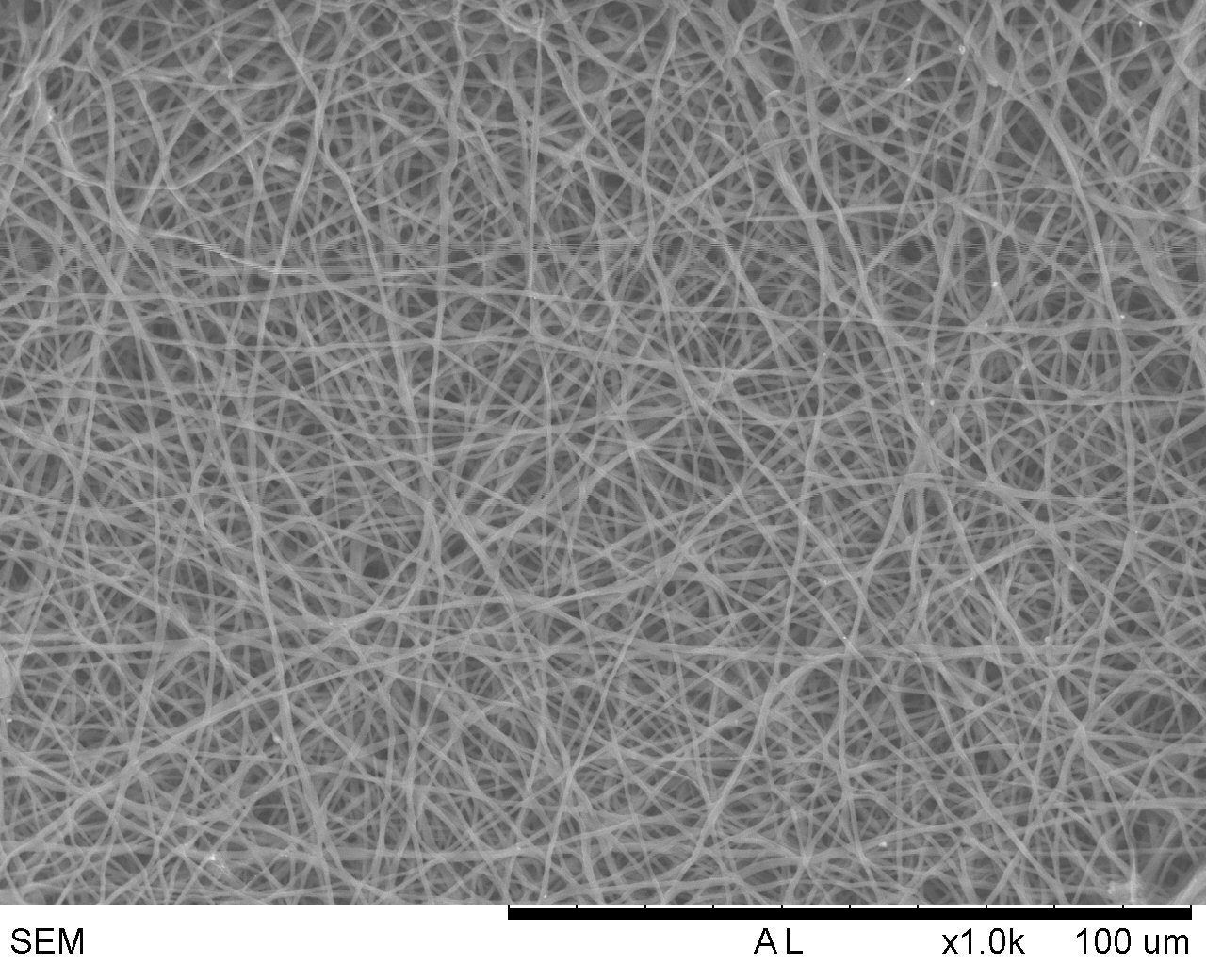

Supplement: Dataset S5 [file peerj-07-6986-s005.zip › SEM/PU Canola Neem oil/SEM(x1.0k).jpg]

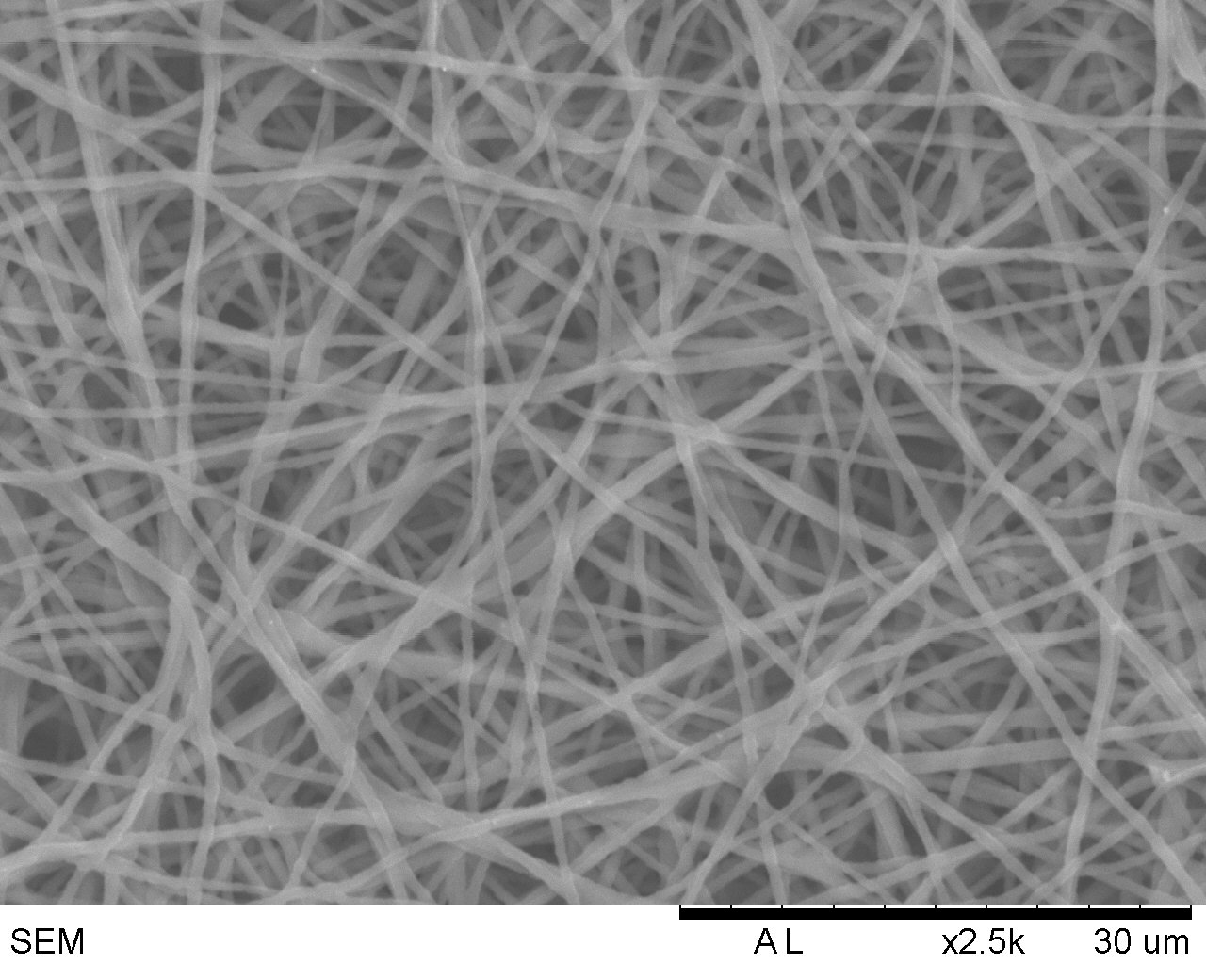

Supplement: Dataset S5 [file peerj-07-6986-s005.zip › SEM/PU Canola Neem oil/SEM(x2.5k).jpg]

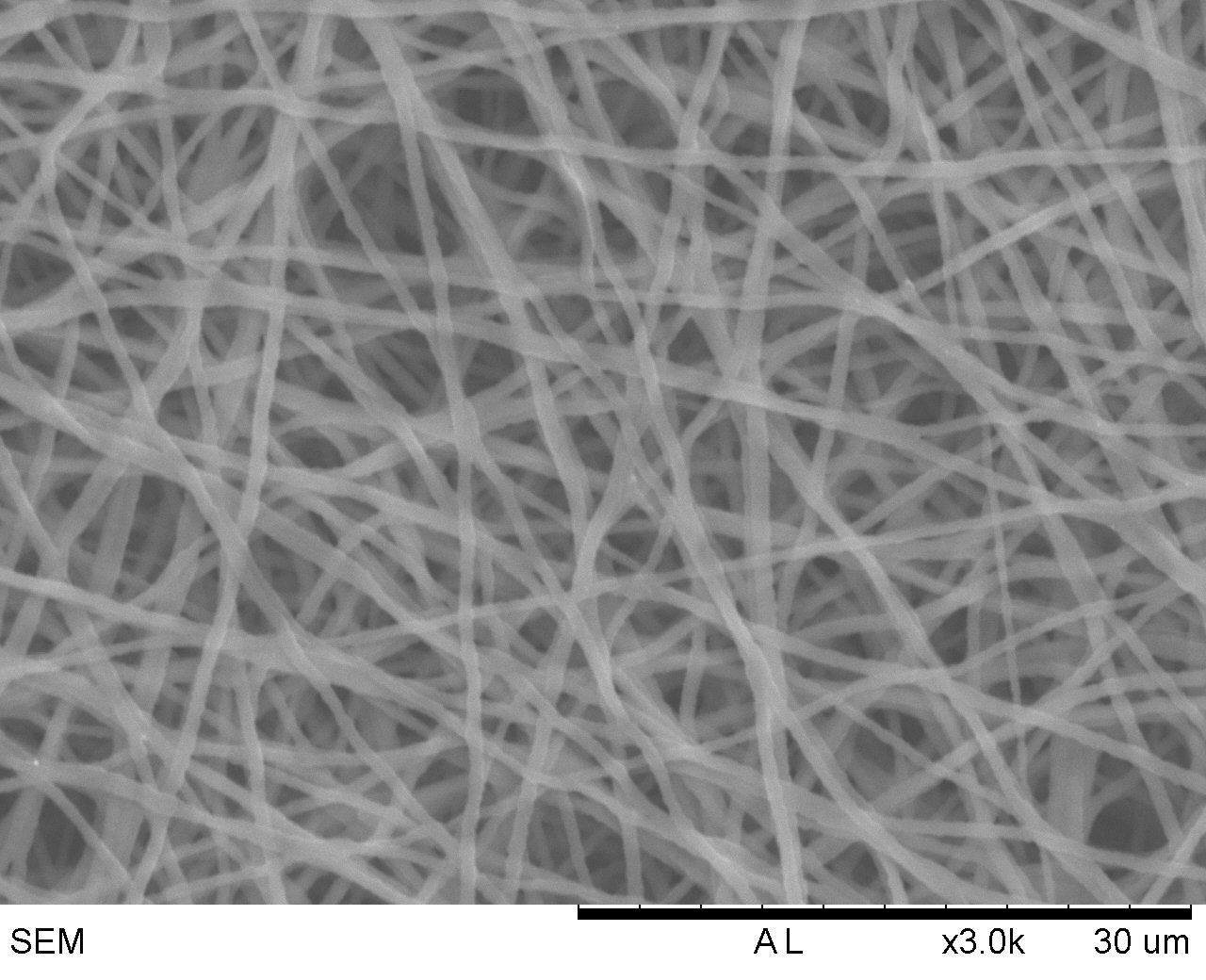

Supplement: Dataset S5 [file peerj-07-6986-s005.zip › SEM/PU Canola Neem oil/SEM(x3.0k).jpg]

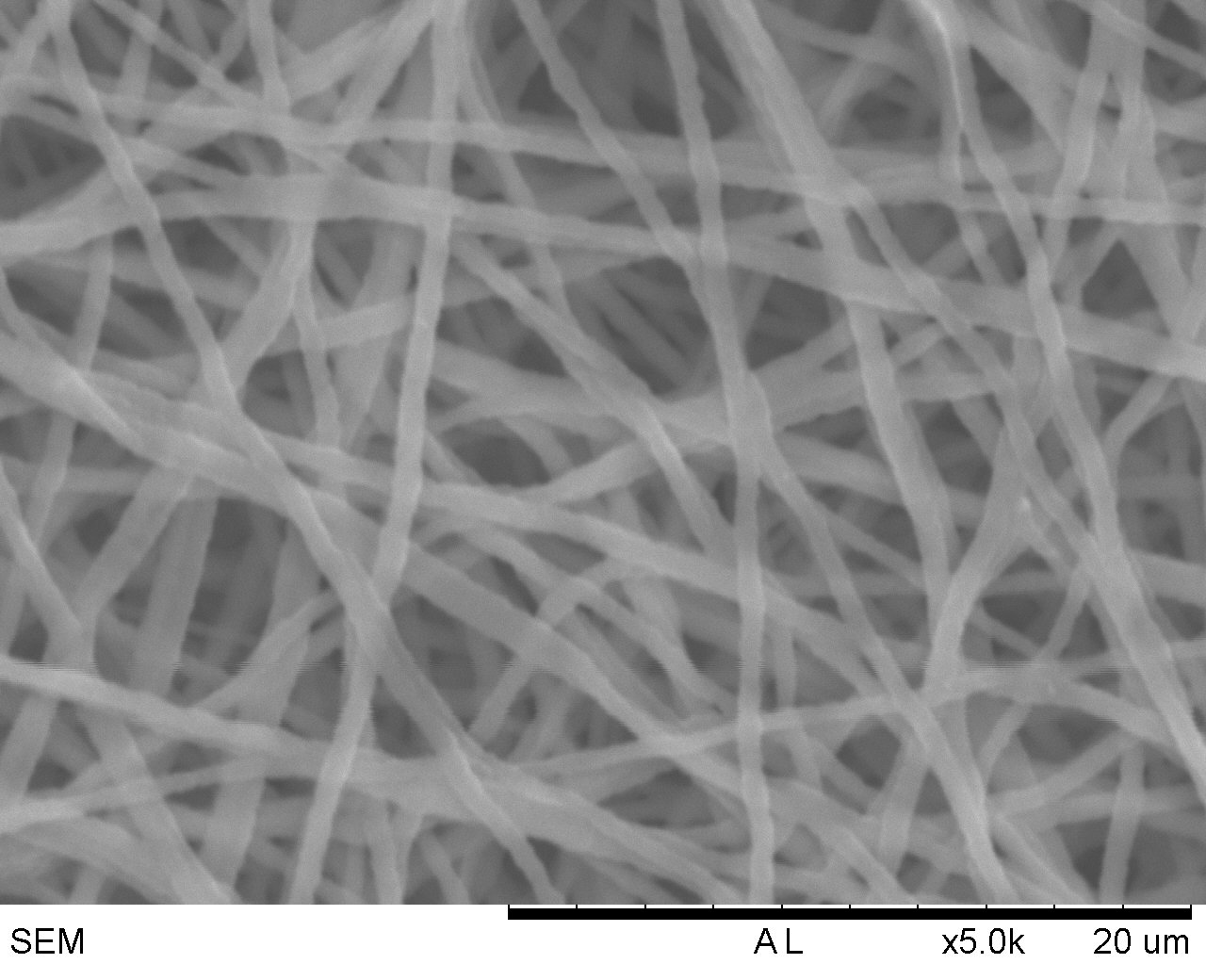

Supplement: Dataset S5 [file peerj-07-6986-s005.zip › SEM/PU Canola Neem oil/SEM(x5.0k).jpg]

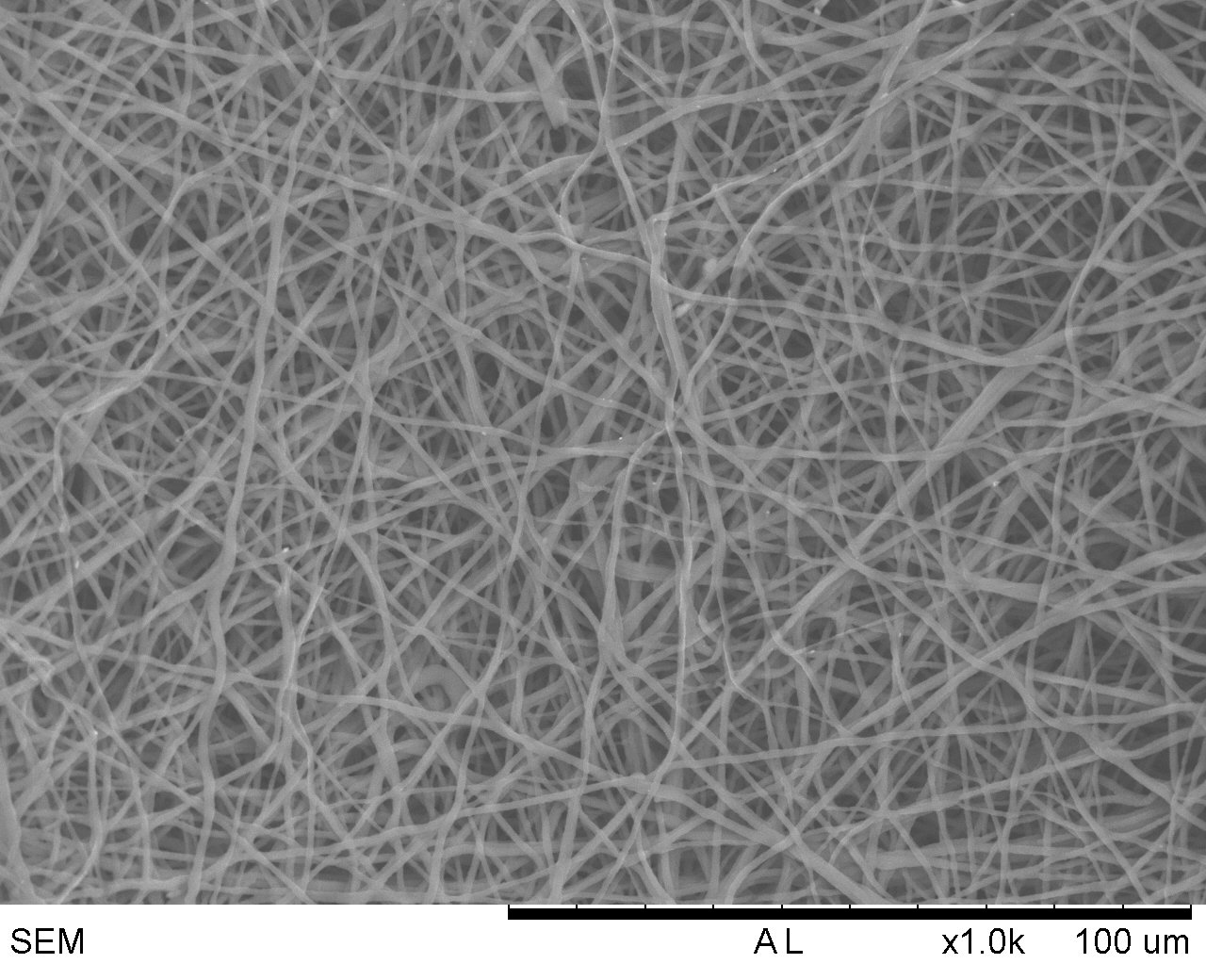

Supplement: Dataset S5 [file peerj-07-6986-s005.zip › SEM/PU Canola Oil/SEM(x1.0k).jpg]

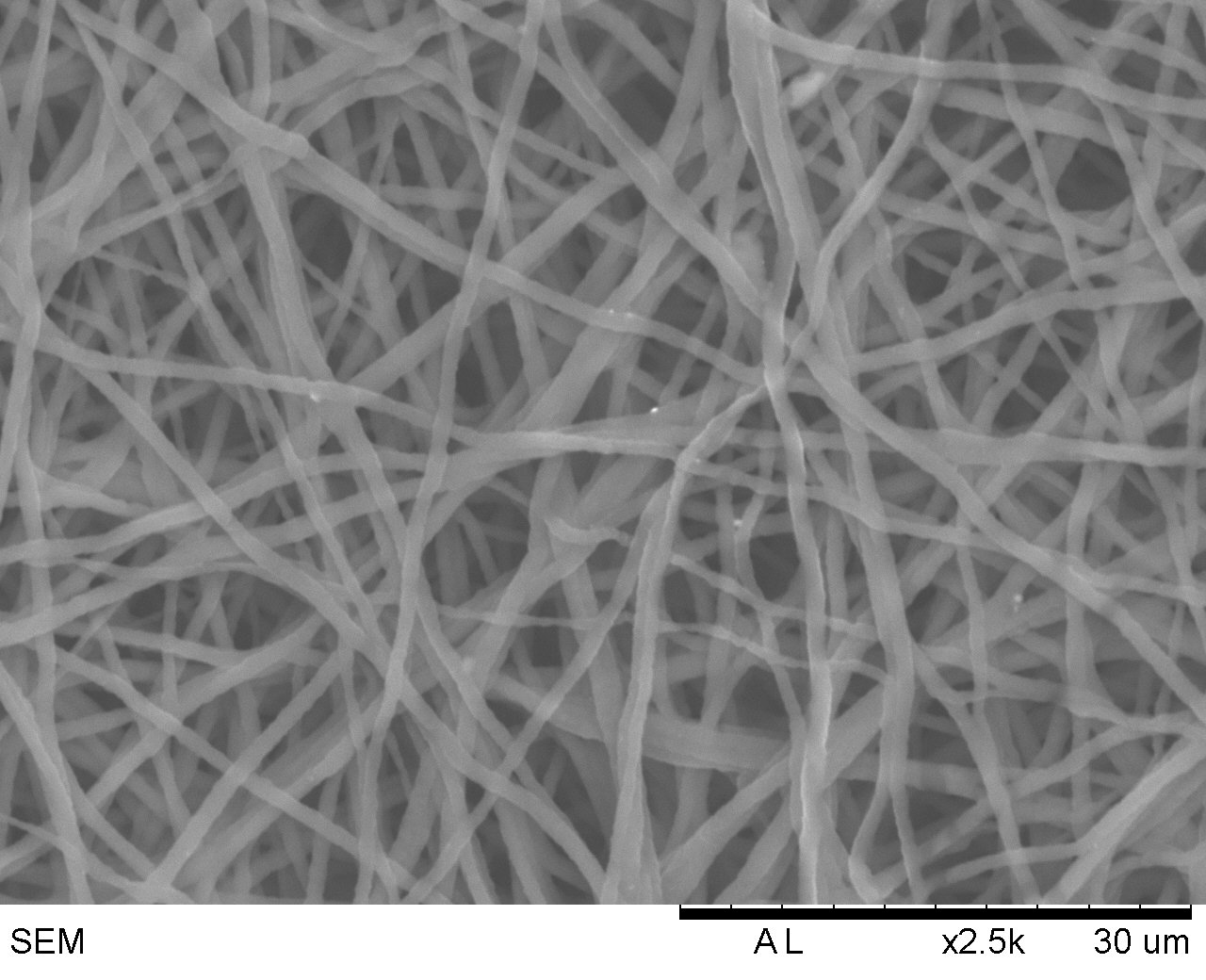

Supplement: Dataset S5 [file peerj-07-6986-s005.zip › SEM/PU Canola Oil/SEM(x2.5k).jpg]

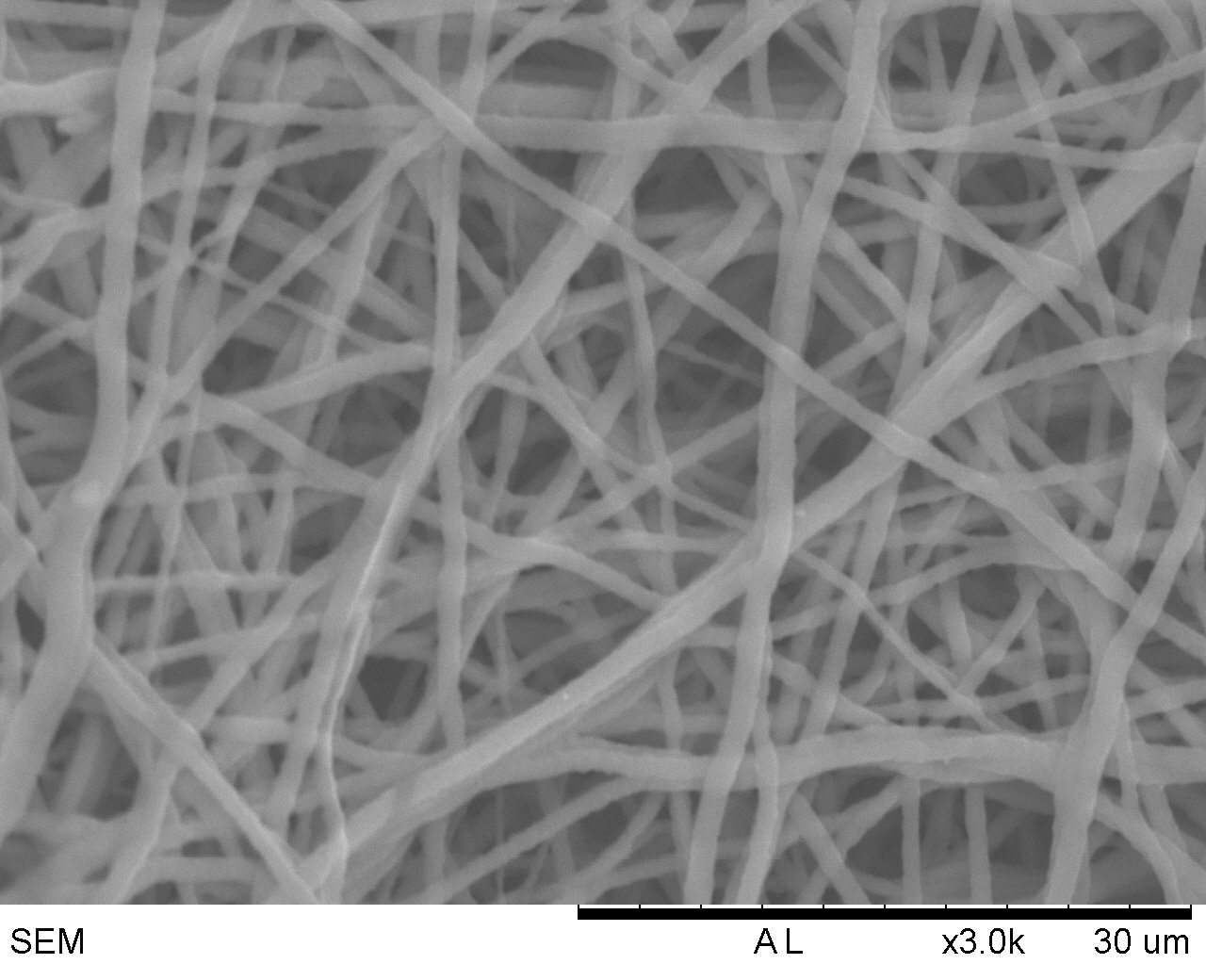

Supplement: Dataset S5 [file peerj-07-6986-s005.zip › SEM/PU Canola Oil/SEM(x3.0k).jpg]

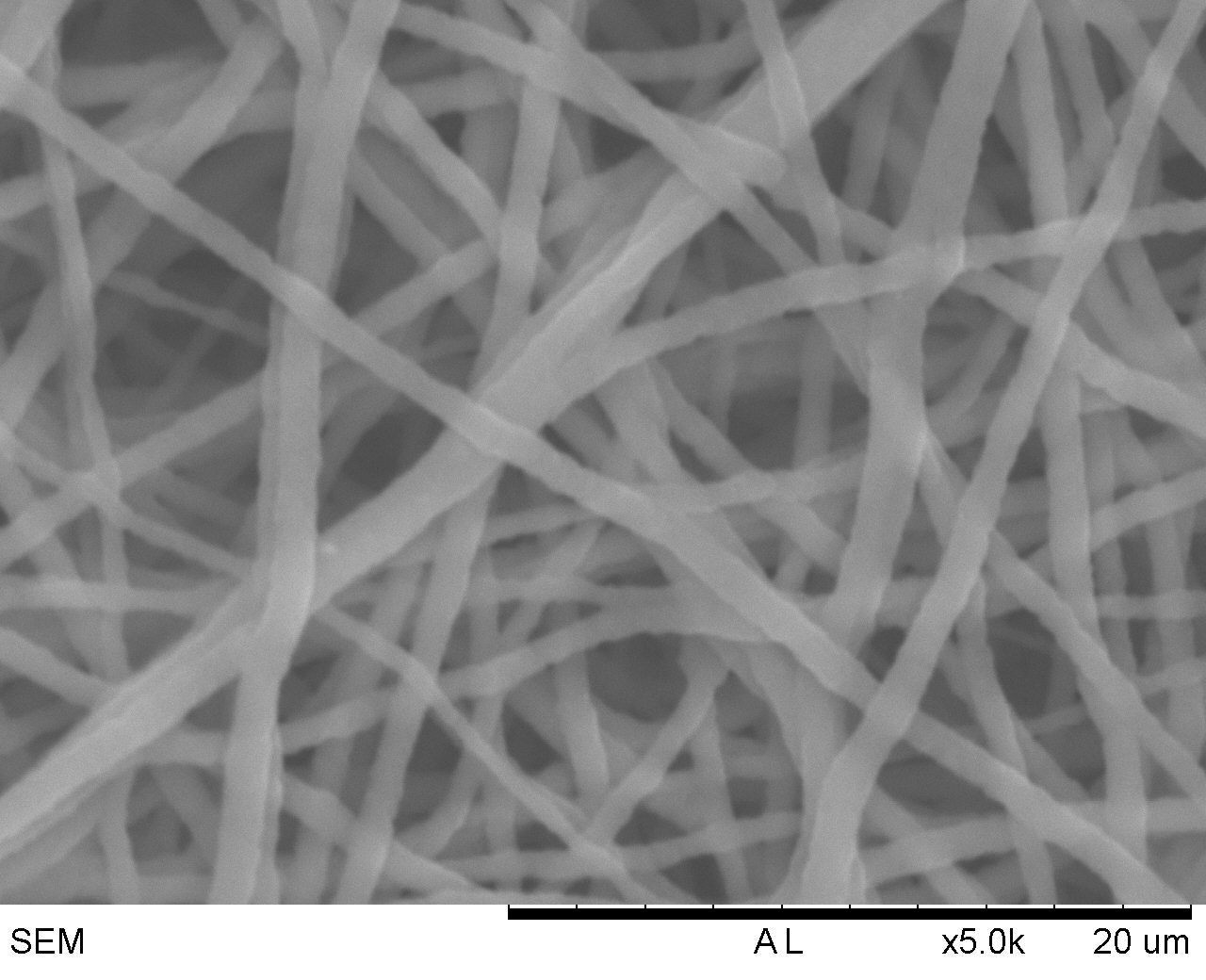

Supplement: Dataset S5 [file peerj-07-6986-s005.zip › SEM/PU Canola Oil/SEM(x5.0k).jpg]

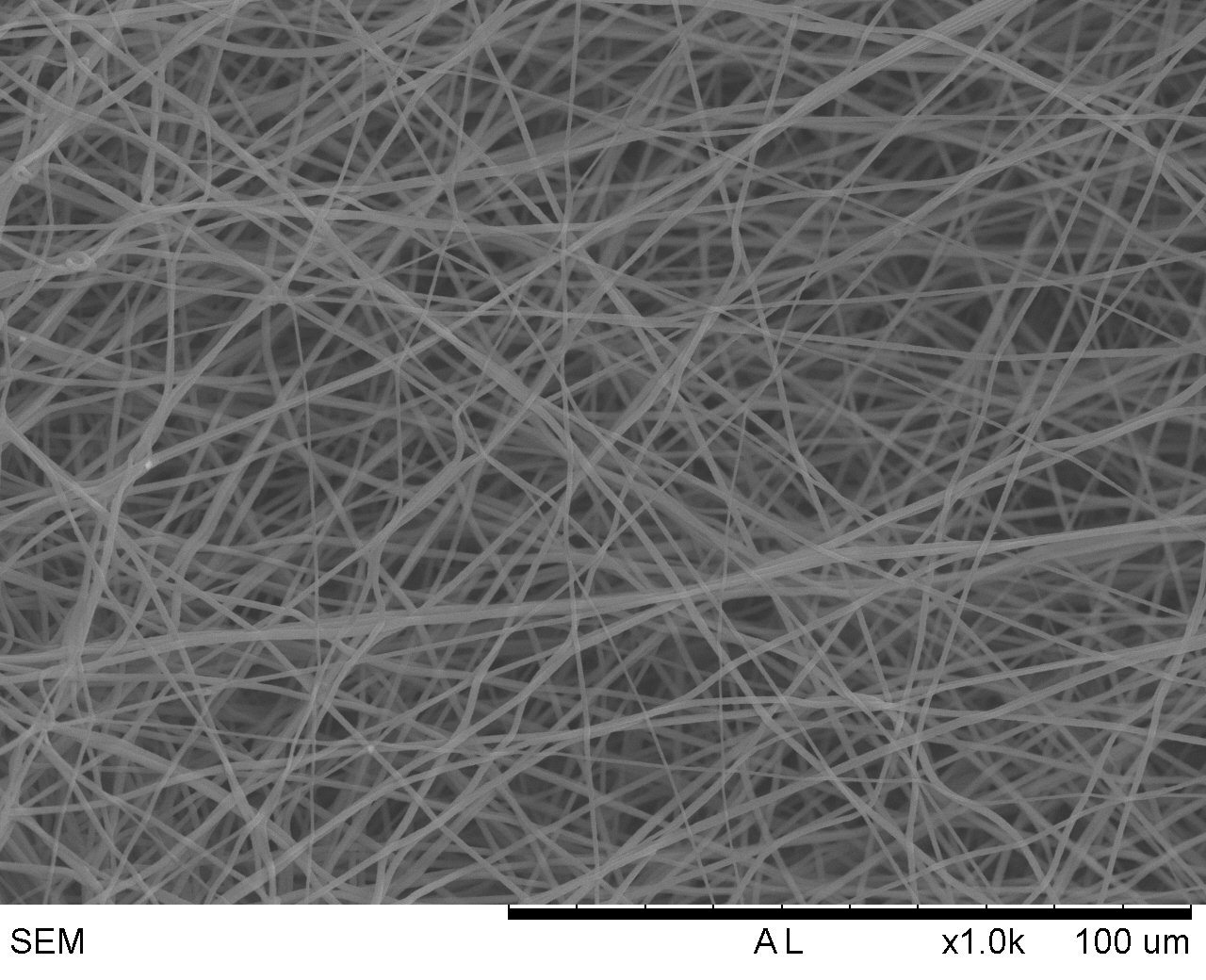

Supplement: Dataset S5 [file peerj-07-6986-s005.zip › SEM/pure Pu 9%/SEM(x1.0k).jpg]

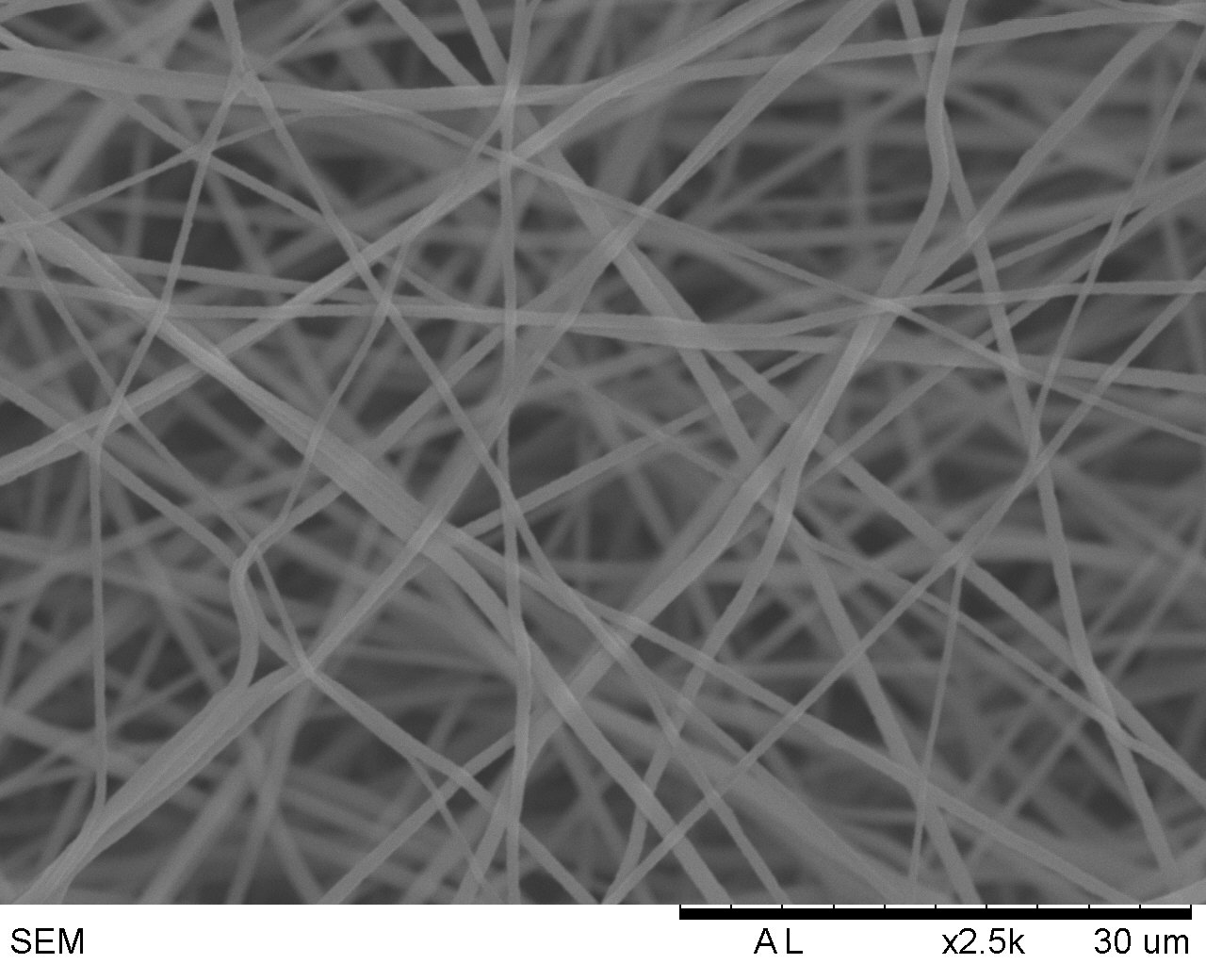

Supplement: Dataset S5 [file peerj-07-6986-s005.zip › SEM/pure Pu 9%/SEM(x2.5k).jpg]

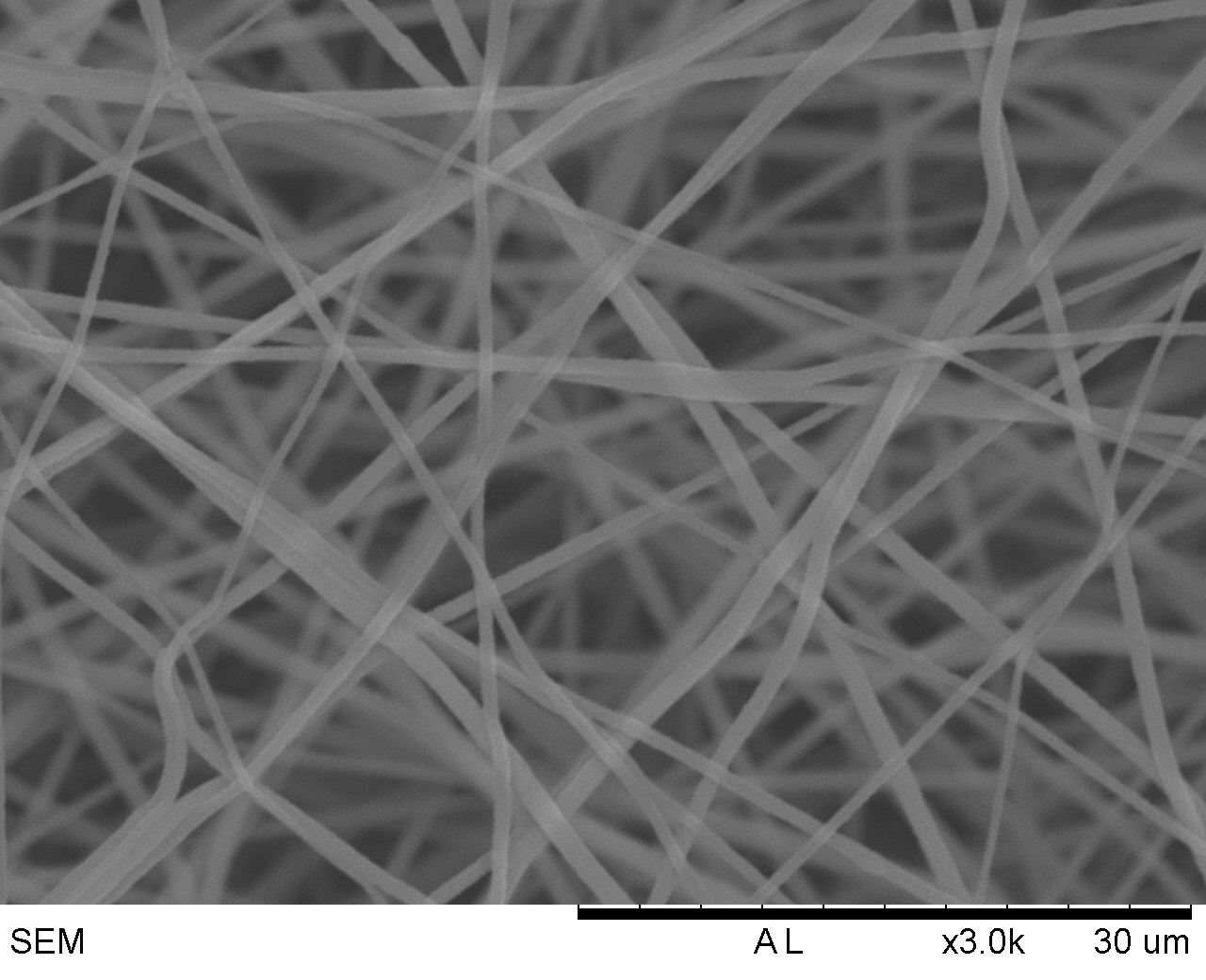

Supplement: Dataset S5 [file peerj-07-6986-s005.zip › SEM/pure Pu 9%/SEM(x3.0k).jpg]

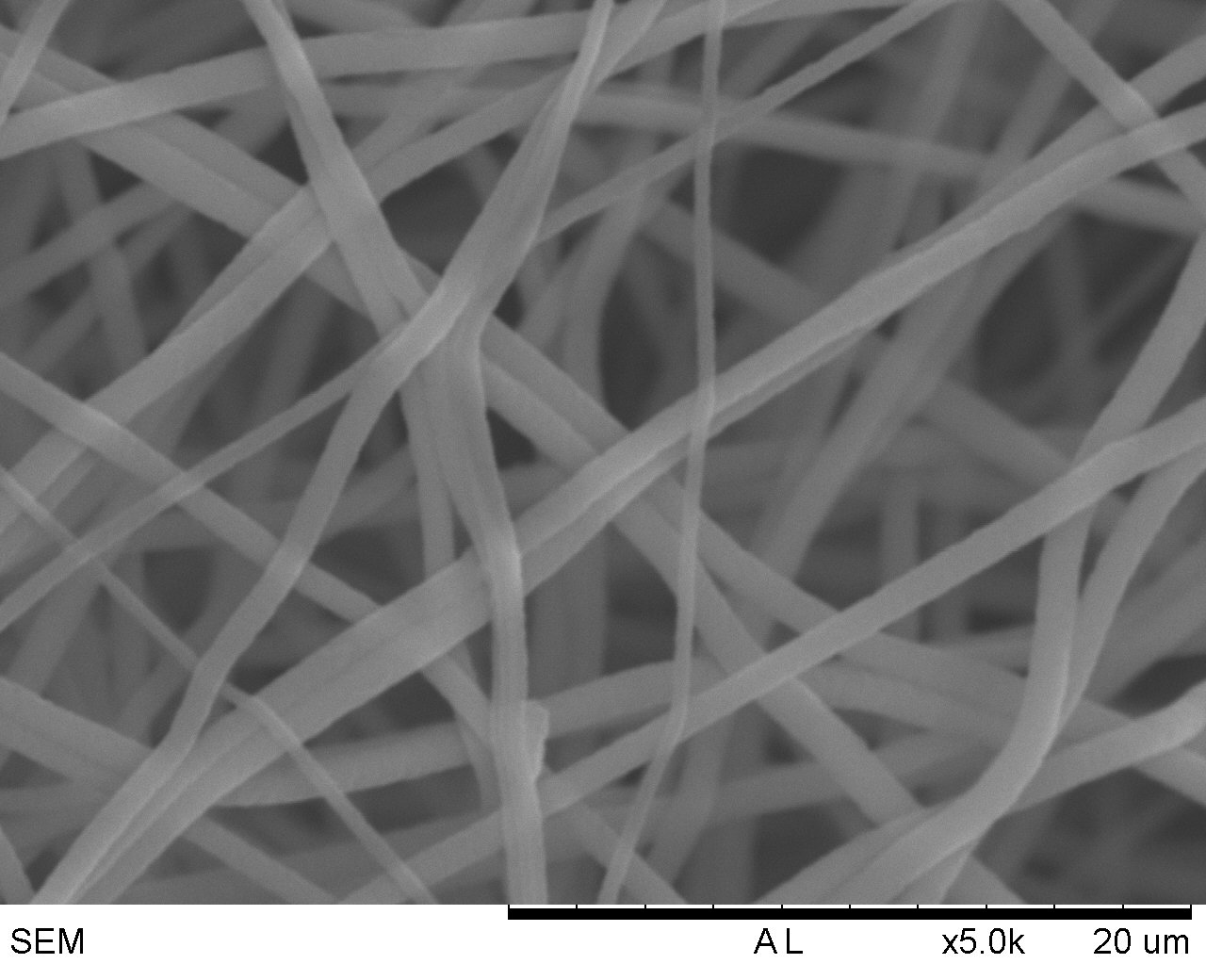

Supplement: Dataset S5 [file peerj-07-6986-s005.zip › SEM/pure Pu 9%/SEM(x5.0k).jpg]
